# Seasonal Variation in In Hospite but Not Free-Living, Symbiodiniaceae Communities Around Hainan Island, China

**DOI:** 10.3390/microorganisms13081958

**Published:** 2025-08-21

**Authors:** Tinghan Yang, Zhao Qi, Haihua Wang, Pengfei Zheng, Shuh-Ji Kao, Xiaoping Diao

**Affiliations:** 1 College of Ocean and Earth Sciences, Xiamen University, Xiamen 361102, China; yangtinghan@126.com; 2 State Key Laboratory of Marine Resource Utilization in South China Sea, Hainan University, Haikou 570228, China; 3 Department of Animal Ecology & Systematics, Justus Liebig University, 35392 Giessen, Germany; 4 College of Life Sciences, Hainan Normal University, Haikou 571158, China; 5 Fourth Institute of Oceanography, Ministry of Natural Resources, Beihai 536000, China

**Keywords:** Symbiodiniaceae community structure, seasonal variation, adaptability regulation, symbiont shuffling, symbiont interaction

## Abstract

Coral reefs are increasingly threatened by global climate change, and mass bleaching and mortality events caused by elevated seawater temperature have led to coral loss worldwide. Hainan Island hosts extensive coral reef ecosystems in China, yet seasonal variation in Symbiodiniaceae communities within this region remains insufficiently understood. We aimed to investigate the temperature-driven adaptability regulation of the symbiotic Symbiodiniaceae community in reef-building corals, focusing on the environmental adaptive changes in its community structure in coral reefs between cold (23.6–24.6 °C) and warm (28.2–30.6 °C) months. Symbiodiniaceae shuffling and rare genotype turnover were discovered in adaptability variations in the symbiotic Symbiodiniaceae community between two months. Symbiodiniaceae genetic diversity increased during warm months, primarily due to temporal turnover of rare genotypes within the *Cladocopium* and *Durusdinium* genera. Coral *Favites*, *Galaxea*, and *Porites* exhibited the shuffling of Symbiodiniaceae between tolerant *Durusdinium* and sensitive *Cladocopium*. Symbiodiniaceae interactions in *G. fascicularis* and *P. lutea* exhibited the highest levels of stability with the increase in temperature, whereas the interactions in *A. digitifera* and *P. damicornis* showed the lowest levels of stability. Rare genotypes functioned as central hubs and important roles within Symbiodiniaceae communities, exhibiting minimal responsiveness to temperature fluctuations while maintaining community structural stability. The temperature-driven adaptability regulation of symbiotic Symbiodiniaceae could be achieved by Symbiodiniaceae shuffling and rare genotype turnover. The process might be aggravated by concurrent adverse factors, including elevated salinity, pollution, and anthropogenic disturbance. These findings provide insights into how the Symbiodiniaceae community influences the adaptation and resilience of coral hosts to temperature fluctuations in coral reefs. Furthermore, they may contribute to assessing the reef-building coral’s capacity to withstand environmental stressors associated with global climate change.

## 1. Introduction

Coral reefs are among the most productive and biodiverse marine ecosystems [1]. Scleractinian corals establish an essential symbiotic association with photosynthetic dinoflagellates of the family Symbiodiniaceae, a partnership fundamental to the persistence of coral reefs and their associated communities [2,3,4]. Symbiodiniaceae supply reef-building corals with photosynthates necessary for calcium carbonate skeletogenesis and the development of extensive reef frameworks, which underpin the success of reef-building corals as habitat engineers in coral reef ecosystems [5,6,7]. However, coral reefs face escalating threats from global climate change, particularly mass bleaching events and mortality caused by elevated sea surface temperature (SST), which have caused extensive coral loss worldwide [8,9]. Critically, the long-term response and regulation of coral reefs to climate change depends on the ability of coral-Symbiodiniaceae symbiosis to acclimate to environmental changes [10,11]. Consequently, understanding the mechanisms that regulate the adaptability of endosymbiotic Symbiodiniaceae in reef-building corals under varying marine environmental conditions, such as variations in seawater temperature, has garnered substantial attention from conservation biologists, particularly in the context of global climate change [12,13,14].

Symbiodiniaceae community structure within corals serves as a robust indicator of host health and stress tolerance [1]. This community is integral to the holobiont’s fitness (holobiont denotes an ecological unit comprising a host and its associated microorganisms), acclimatization, adaptability, and survival, both under homeostatic and adverse conditions [15]. The specific effects of Symbiodiniaceae composition, abundance, and diversity on coral acclimatization and adaptability remain poorly understood [16,17]. Substantial evidence confirms that the Symbiodiniaceae community maintains nutrient equilibrium and promotes community interactions and stability within coral holobionts [18,19,20,21], thereby maintaining symbiotic homeostasis under thermal stress [5,22,23,24]. However, the effects of different Symbiodiniaceae types in host coral remain poorly understood [25]. It is only in recent years that the role of different Symbiodiniaceae types in influencing the nutritional capacity and trophic plasticity of coral hosts under varying temperature conditions has been investigated [5,26]. The temperature effects on Symbiodiniaceae community structure constitute a long-standing research focus [7,27,28], whereas the adaptability regulation of the Symbiodiniaceae community structure and the roles of various Symbiodiniaceae types in host acclimatization warrant deeper investigation. Despite established knowledge of Symbiodiniaceae’s role in coral-symbiont communication [20,24], the dynamics of Symbiodiniaceae community interactions and stability across host species during environmental temperature changes remain limited [21,29]. Furthermore, investigations linking Symbiodiniaceae regulatory adaptation and genotypic interactions to reef-building coral resilience remain scarce [8,30].

The adaptive capacity of reef-building corals under climate change depends partly on genetic variation in their endosymbiotic symbionts and ambient environmental parameters critical for sustaining coral-Symbiodiniaceae mutualism [31,32,33]. Symbiodiniaceae possess generation times orders of magnitude faster than their host corals, conferring greater adaptive potential to environmental perturbations [34,35]. Differential physiological traits among Symbiodiniaceae genera/species, including intrinsic adaptive capacity and stress tolerance thresholds, significantly determine coral holobiont adaptivity [22,36,37]. This partly confers considerable disparities in environmental tolerance among different coral colonies and species [38]. Previous reports evidenced that corals associating with *Durusdinium* spp. exhibit superior thermal tolerance compared to those hosting *Cladocopium* species [39,40,41]. For example, both cold and hot seawater events, increasingly frequent and more severe under climate change, easily lead to coral bleaching and mortality primarily through disruption of the coral-Symbiodiniaceae symbiosis [42,43]. Multiple studies observed that Symbiodiniaceae communities exhibit adaptive restructuring in response to environmental stressors, thereby modulating coral acclimatization [12,32,44]. For example, bleached corals might recover through restructuring of thermotolerant *Durusdinium* strains [45]. Most host corals do not typically shuffle their dominant Symbiodiniaceae to different genera under stable conditions, unless environmental stressors are severe enough to trigger changes in the symbiont community [46,47]. Although the background Symbiodiniaceae in corals may shuffle to dominance with associated changes under such environmental conditions, their functional roles to the coral holobionts or the overall Symbiodiniaceae community structure remain poorly understood [48].

While the symbiont shuffling and switching hypothesis remains contentious, some studies suggest that coral holobionts may rapidly and efficiently acclimate via symbiont reorganization [31,33,49], involving (1) shuffling the relative abundance of existing Symbiodiniaceae genotypes and/or (2) switching to novel genotypes restructured from the surrounding environment—predominantly from adjacent seawater [50]. However, the influence of temperature-driven regulation of Symbiodiniaceae community structure on different host corals under natural environmental conditions remains insufficiently investigated [33,41]. In this study, we investigate seasonal changes in the community structure, stability, and interactions of both in hospite and free-living Symbiodiniaceae in reef-building corals. Specifically, we examine how environmental fluctuations between cold and warm months influence symbiont composition and adaptability regulation under natural environmental conditions. This research aims to elucidate the mechanisms by which coral holobionts respond to thermal stress through symbiont community restructuring.

## 2. Materials and Methods

### 2.1. Study Location

**Figure 1:**
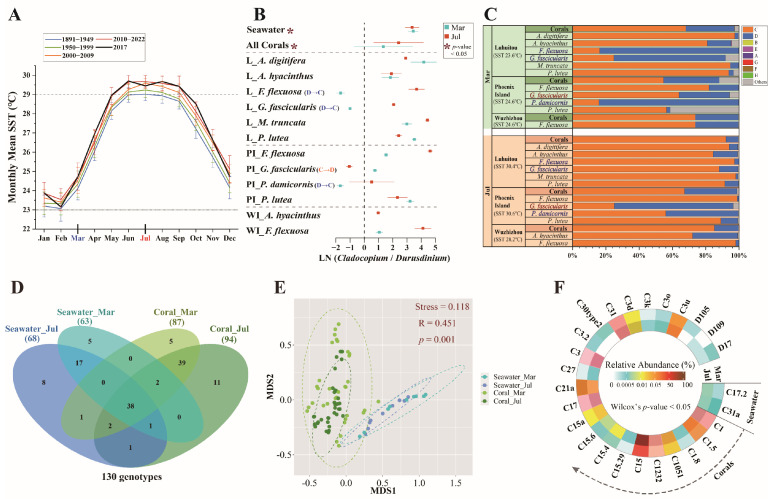
The mean monthly sea surface temperature (SST) in Hainan Island of China (**A**) and the free-living and in hospite Symbiodiniaceae communities from coral reefs. (**B**) Changes in the relative abundance of *Cladocopium* and *Durusdinium* in March and July as measured by the logarithmic ratio (logarithm to the base 10) (*, Chi-squared test, *p*-values < 0.05). The letters L, PI, and WI represent Luhuitou, Phoenix Island, and Wuzhizhou Island, respectively. The D → C and C → D denote the shuffling between *Cladocopium* and *Durusdinium* dominance from March to July. (**C**) The community distribution of the genus levels of endosymbiotic Symbiodiniaceae within various coral species at the sampling sites. (**D**) The Venn diagram showing the number of genotypes in seawater and corals. (**E**) The non-metric multidimensional scaling (NMDS) ordination based on Bray–Curtis dissimilarity matrix of the Symbiodiniaceae community. (**F**) Comparison for the relative abundance of genotypes with significant changes (Wilcox’s test, *p*-values < 0.05) between March and July.

Luhuitou, Phoenix Island, and Wuzhizhou Island are part of the Sanya Coral Reef National Nature Reserve (SCRNNR, 18°10′30″ N–18°15′30″ N, 109°20′50″ E–109°40′30″ E), which is a significant area for coral reef distribution in Sanya of Hainan Island. Hainan Island (108°37′ N–111°03′ N, 18°10′ E–20°10′ E) is situated in the southernmost region of China and is characterized by an abundance of coral reefs. The island has a typical wet season from May to October and a dry season from January to April and November to December. Historical SST data for Hainan Island shows that mean monthly SST is higher in April–October and lower in January–March and November–December (data from the National Oceanic and Atmospheric Administration, https://psl.noaa.gov the access data is 8 December 2022), with a temperature difference of 4–5 °C (Figure 1A). In 2017, mean monthly SST in March and July were recorded at 24.7 °C and 29.5 °C, respectively, showcasing a difference of 4.8 °C (Table S1). Sanya, located at the southernmost tip of Hainan Island, is a renowned international tourist city with an average annual SST ranging from 20 °C to 33 °C (data from China Meteorological Data Network, http://data.cma.cn, the access data is 8 December 2022). The city experiences its peak tourist season in January–April and October–December, with the off-season for tourism in May–September. Despite boasting the rich diversity of scleractinian coral species and the extensive coral coverage in China, the fringing reefs along the coast of Hainan Island have seen a significant decline of at least 80% of their coral cover over the past three decades [51]. Large-scale global thermal bleaching caused by climate change has adversely impacted coral reef ecosystems and the healthy growth of corals on Hainan Island, particularly within SCRNNR [52,53,54]. Coral bleaching heat stress was generally from May to October, and the bleaching alert level in Hainan was increasing year by year (Figure S1A). The alert level of coral bleaching heat stress in March was no stress, while it was bleaching watch and alert level 1 in July 2017.

### 2.2. Sample Collection and Environmental Factors Determination

Sampling was conducted in March and July. Because these months, respectively, represent the peak tourism season with seasonally low SST and “No Stress” coral bleaching alert levels, and the off-peak tourism period with seasonally high SST and the occurrence of “Alert Level 1” in Hainan. Coral sampling encompassed seven dominant species (*Acropora digitifera*, *Pocillopora damicornis*, *Porites lutea*, *Galaxea fascicularis*, *Favites flexuosa*, *Montipora truncata*, and *Acropora hyacinthus*) that were evenly sampled across sites and months and evenly distributed across sampling months. Each coral species was represented by at least 3 fragments per site per month. A total of 69 healthy coral fragments (2–3 cm in length or width, collected at about 1.5–3 m in depth), coral-surrounding surface seawater (collected at 50 cm in depth, *n* = 3 per site × 3 L per sample), and coral ambient sediments (collected at 0–3 cm in depth, *n* = 3 per site × 100 mL per sample) were synchronously sampled during March and July 2017 at the three sites of SCRNNR. The sampling locations were recorded using the global positioning system (Figure S1). Coral fragments were collected by SCUBA divers using pliers from isolated clusters of distinct coral colonies along linear transects spanning at least 5 m. These fragments were cleaned with artificial sterile seawater with a salinity of 35‰ to avoid contamination by free-living Symbiodiniaceae in seawater. All coral sample collections were conducted in accordance with the guidelines and permitted by the Tropical Marine Biological Research Station in Hainan, the Hainan South China Sea Institute of Tropical Oceanography, and the Hainan Province Modern Marine Ranching Engineering Research Center. We have complied with all relevant ethical regulations for animal use. Coral species were morphologically identified with the help of reference books and experts from the South China Sea Institute of Oceanology, Chinese Academy of Sciences, and Hainan South China Sea Institute of Tropical Oceanography. Coral samples from seven species belonging to six genera were collected due to their universality or preponderance in the study region (Table S2). A total of 18 samples for free-living Symbiodiniaceae in seawater (2 L per sample) were collected by filtering through 0.22 μm polycarbonate membranes (Millipore, Burlington, MA, USA). Upon collection, the above coral samples and membranes were immediately frozen in liquid nitrogen and stored at −80 °C back in the laboratory. Coral samples were ground using liquid nitrogen, and membranes with plankton cells were cut into pieces with sterile scissors. These samples were transferred into sterile tubes with DNA lysis buffer and stored at −20 °C for DNA extraction. Intensive maritime operations and transportation generate substantial crude oil and combustion-derived residues [55]. Polycyclic aromatic hydrocarbons (PAHs) are a primary component of such pollutants and are widely distributed in the coral reefs along the coast [56,57], impacting coral health [58]. Consequently, we quantified PAH concentrations in seawater and sediments. Specific determination methods of environmental parameters [59] and sixteen polycyclic aromatic hydrocarbons (∑PAHs) [60] are described in the Supplementary Materials.

### 2.3. DNA Extraction and Amplification of Symbiodiniaceae ITS2 rDNA

The above samples were incubated at 56 °C with proteinase K (Solarbio, Beijing, China) for about three days, with the extended incubation period intended to maximize cell lysis. Then recalcitrant cells that remained unbroken at the end of incubation were mechanically homogenized using the Fastprep^®^-24 Sample Preparation System (MP Biomedicals, Irvine, CA, USA) with bead-beating (~3:1 mixture of 0.5 mm and 0.1 mm diameter ceramic beads) as previously reported [61]. Next, total genomic DNA was extracted using the cetyltrimethylammonium bromide (CTAB) method and purified with the Zymo DNA Clean & Concentrator kit (Zymo Research, Irvine, CA, USA) following Yuan, Li and Lin [61]. DNA integrity was verified by 1% agarose gel electrophoresis, revealing distinct high-molecular-weight bands without degradation. NanoDrop 2100 spectrophotometry (Thermo Fisher Scientific, Waltham, MA, USA) verified DNA purity (A260/A280 ratios: 1.8–2.0; A260/A230: 1.8–2.2) across all samples. The internal transcribed spacer 2 (ITS2) region of Symbiodiniaceae nuclear ribosomal DNA was amplified through polymerase chain reaction (PCR) with the ITS2-forward (5′-GTGAATTGCAGAACTCCGTG-3′) and ITS2-reverse (5′-CCTCCGCTTACTTATATGCTT-3′) primers [62]. The PCR amplification was performed with the following program: 94 °C for 5 min, followed by 30 cycles of 95 °C for 30 s, 52 °C for 30 s, and 72 °C for 60 s, with a final extension at 72 °C for 10 min. Detailed amplification and purification protocols are provided in Supplementary Materials.

### 2.4. Sequencing and Bioinformatic Analysis

The purified PCR products were combined in equal proportions and subsequently sequenced on an Illumina HiSeq2500 platform at Novogene Technology Co., Ltd. (Beijing, China). The raw sequences were obtained by splicing all sequences with the barcode and primer sequences truncated using FLASH (v1.2.7) [63]. Strict quality control and sequence filtration were conducted using QIIME (v1.9.1) [64]. This included demultiplexing and quality control steps such as filtering out sequences with low read quality (<20) and short reads (less than 75% of raw sequences). Raw sequences underwent quality filtering and chimera removal using MOTHUR (v1.45.3) and UCHIME (v4.2.40) [65,66]. Subsequently, the sequences were de novo clustered at 97% similarity and categorized into operational taxonomic units (OTUs) using the UCLUST algorithm. This threshold was selected to minimize intragenomic ITS2 variation (known to average 2–3% within Symbiodiniaceae genomes) [67] and balance biological relevance against artificial inflation from sequencing errors [68]. Representative sequences were aligned to the Symbiodiniaceae ITS2 database for the highest identity by BLAST (v2.13.0). Singleton OTUs (*n* = 1 across all samples) were removed to reduce spurious sequences, while preserving rare biosphere diversity through (1) retention of OTUs appearing in ≥2 samples; (2) validation against rarefaction curves showing asymptotic diversity accumulation (Figure S2); and (3) comparative analysis confirming retained OTUs represented genuine rare genotypes (e.g., presence in negative controls < 0.001%). Alpha diversity (Chao1, Shannon) was calculated from subsampled OTU tables. Species accumulation and rarefaction curves confirmed adequate sequencing depth (Figure S2). The Symbiodiniaceae ITS2 database is a non-redundant database that integrates published Symbiodiniaceae ITS2 sequences [62,67,69,70,71,72] and the GeoSymbio database [73]. The raw ITS2 sequences have been deposited in the NCBI Sequence Read Archive Database under accession number PRJNA1148647.

### 2.5. Statistical Analysis and Visualization

Symbiodiniaceae genotypes were categorized based on relative abundance: >1% as ‘abundant types’, <0.01% as ‘rare types’, and 0.01–0.1% as ‘intermediate types’ [74,75]. The study compared the abundance of Symbiodiniaceae genotypes in corals and seawater using Wilcoxon rank-sum and chi-squared tests. Co-occurrence networks of Symbiodiniaceae genotypes in seawater and corals were established using Cytoscape 3.9.3, involving basic configuration, permutation, bootstrapping, and network restoration from random files in CoNet. Pairwise associations among Symbiodiniaceae genotypes were analyzed using correlation measures (Pearson and Spearman) and dissimilarity measures (Bray–Curtis and Kullback–Leibler) with initial thresholds of 1000 top and bottom edges. Isolated nodes were excluded from the network, and the resulting network graphs were visualized using Gephi 0.9. Network complexity was quantified by the ratio of edges to nodes, while network vulnerability was assessed by the maximal vulnerability of nodes. Individual node vulnerability reflects its contribution to global efficiency. Complexity and vulnerability were used as indirect indicators to evaluate the adaptive capacity of Symbiodiniaceae communities in response to future environmental change. Compositional stability of the network was applied to evaluate the change in Symbiodiniaceae community structure over time, providing a measure of the community’s resilience against disturbances or temporal fluctuations. Network vulnerability and compositional stability were calculated via R (version 4.2.1) software, following the methodology described by Yuan et al. [76]. A circular maximum likelihood phylogenetic tree was constructed to visualize the relative abundance of Symbiodiniaceae using MEGA X 10.2.6 and Interactive Tree of Life (iTOL v7). The correlation between environmental factors and Symbiodiniaceae communities was analyzed by Pearson correlation analysis and Mantel tests. Non-metric multidimensional scaling (NMDS) based on Bray–Curtis distance matrices was accomplished to explore differences in Symbiodiniaceae communities in corals and seawater between March and July using R with the vegan (v 2.6-4) package. Decision curve analysis (DCA) was conducted prior to redundancy analysis (RDA) to estimate the influence of coral reef environmental factors on Symbiodiniaceae communities in both corals and seawater. This confirmed the suitability of linear-based RDA for modeling factor effects on Symbiodiniaceae communities in corals and seawater. Bar charts, scatter charts, box charts, and heat charts were drawn using Origin 2022b software and R with ggplot2 (v3.5.1), vegan, and ggcor (v 0.9.8.1) packages.

## 3. Results

### 3.1. Differences in Symbiodiniaceae Genetic Diversity Between Cold and Warm Months

Following quality filtering and chimera removal, a total of 6,787,437 high-quality Symbiodiniaceae sequences were retained, yielding 33,281–95,760 sequences per sample. These sequences identified a total of 130 Symbiodiniaceae genotypes, containing *Cladocopium*, *Durusdinium*, *Breviolum*, *Effrenium*, *Symbiodinium*, *Gerakladium*, *Fugacium*, and Clade H (Table S3). The Chao1 richness index revealed relatively higher Symbiodiniaceae diversity in July than March across sample categories: seawater (July: 1691.6 vs. March: 1656.3), corals (July: 1548.5 vs. March: 1264), and seven coral species (July: 1292.8–2043.7 vs. March: 912.4–1622) (Figure S3A–C). The Shannon diversity index revealed reduced evenness in seawater during July (3.7) compared to March (4.1), whereas corals exhibited increased evenness in July (2.1) compared with March (2.0) (Table S4). Notably, *A. hyacinthus* and *G. fascicularis* showed marginally lower Shannon index values (2.7, 2.1) than March (2.2, 2.8), while other species demonstrated elevated values (July: 1.5–3.3; March: 1.3–2.8).

### 3.2. Variations in Symbiodiniaceae Community Between Cold and Warm Months

Symbiodiniaceae communities in seawater and corals were dominated by *Cladocopium* and *Durusdinium* genera (Figure S4). The relative abundance of *Cladocopium* and *Durusdinium* in seawater increased from 32.86% and 1.2% in March to 47.2% and 1.84% in July (Table S3). *Cladocopium* abundance in corals increased by nearly 20% from March to July, whereas *Durusdinium* reduced by 14.2%. The *Cladocopium*/*Durusdinium* ratio decreased in seawater but increased in corals (Figure 1B). The ratio reductions occurred in *A. digitifera* and *P. lutea* at Luhuitou in July, as well as in *G. fascicularis* and *P. lutea* at Phoenix Island; in contrast, increases were observed in other coral species in comparison to March. In March, *Durusdinium* was dominant (>60% relative abundance) in *F. flexuosa* and *G. fascicularis* at Luhuitou, as well as in *P. damicornis* at Phoenix Island; *Cladocopium* was the predominant genus (>55% relative abundance) in the remaining species (Table S5). By July, *Cladocopium* abundance exceeded 55% in all species except *G. fascicularis* at Phoenix Island. Genus-level shuffling was observed in F. flexuosa and *G. fascicularis* at Luhuitou, which shifted from *Durusdinium* to *Cladocopium* dominance; *P. damicornis* at Phoenix Island showed the same transition, whereas *G. fascicularis* (Phoenix Island) shifted conversely (Figure 1C).

**Figure 2:**
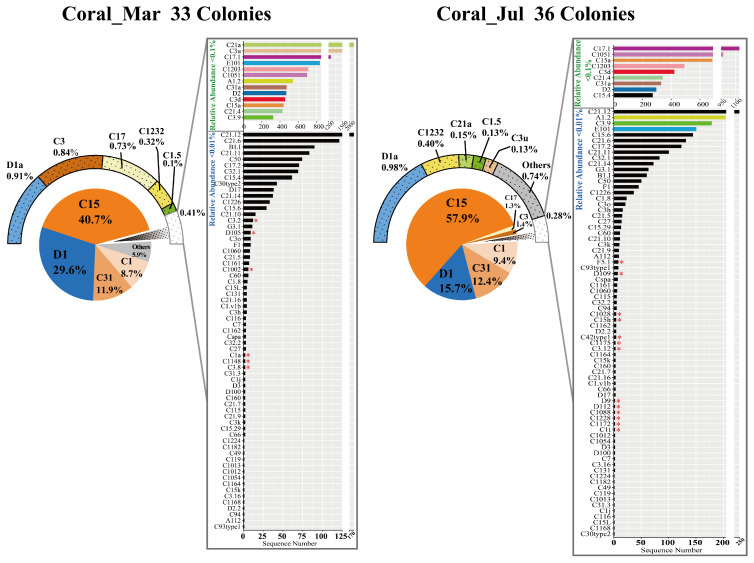
Changes in in hospite Symbiodiniaceae community in March and July. The mean relative abundances between 0.1 and 1% of genotypes were shown in pie charts with a black background, and the relative abundances were displayed in the circular graph. The mean relative abundances < 0.1 of genotypes were displayed in a circular graph with a white background, and sequencing read numbers were exhibited in bar graphs. The genotypes with different color in bar graphs represent their relative abundances > 0.01% in March. The red stars represent undetected genotypes in March/July.

A total of 130 Symbiodiniaceae genotypes, including 75 types in seawater and 99 types in corals, were detected, and there were 38 common genotypes between seawater and corals (Figure 1D). Bray–Curtis dissimilarity analysis revealed significant compositional differences (ANOSIM *R* = 0.451, *p* = 0.001) between seawater and coral communities in both months, with small overlap in March (Figure 1E). Symbiodiniaceae genotype abundances differed significantly (*p* < 0.05) between March and July (Figure 1F). C15 abundance increased by 16.8% in corals during July (Table S6). Abundant types C31 and C1 rose, as well as intermediate types C1232, C21a, C1.5, and C3u (Table S7). In contrast, the abundance of abundant types D1 decreased by 13.7% in July, and intermediate types D2 declined by 1.6 times. Other genotypes in corals also experienced noticeable alterations in abundance, along with shuffling and the difference in rare genotypes detected between cold and warm months (Figure 2). For example, C17 and C3 transferred from previously intermediate types (March: 0.73%, 0.84%) to abundant types (July: 1.26%, 1.35%). Six rare genotypes in March were undetectable by July; conversely, 13 previously undetected rare genotypes emerged during July, comprising predominantly *Cladocopium* (12 genotypes) with four *Durusdinium* genotypes.

The majority of Symbiodiniaceae detected in seawater and corals were members of *Cladocopium*, with 106 genotypes in total, followed by *Durusdinium* with 12 genotypes (Figure S5). Dominant genotypes included C15, C1, and C31; subdominant genotypes comprised D1a (seawater) and D1 (corals). Consistent with genus-level shuffling, D1-dominated communities in *F. flexuosa*, *G. fascicularis* (Luhuitou), and *P. damicornis* (Phoenix Island) in March shifted to C15 and C31 dominance in July (Figure S6). *G. fascicularis* at Phoenix Island showed the opposite shift from C15 to D1 dominance. *A. hyacinthus* at Luhuitou and *F. flexuosa* at Wuzhizhou Island shuffled from C15 and C31 co-dominance in March to C15 monodominance in July (Table S8).

### 3.3. Network Analysis of Genotypes Within Symbiodiniaceae Community

**Figure 3:**
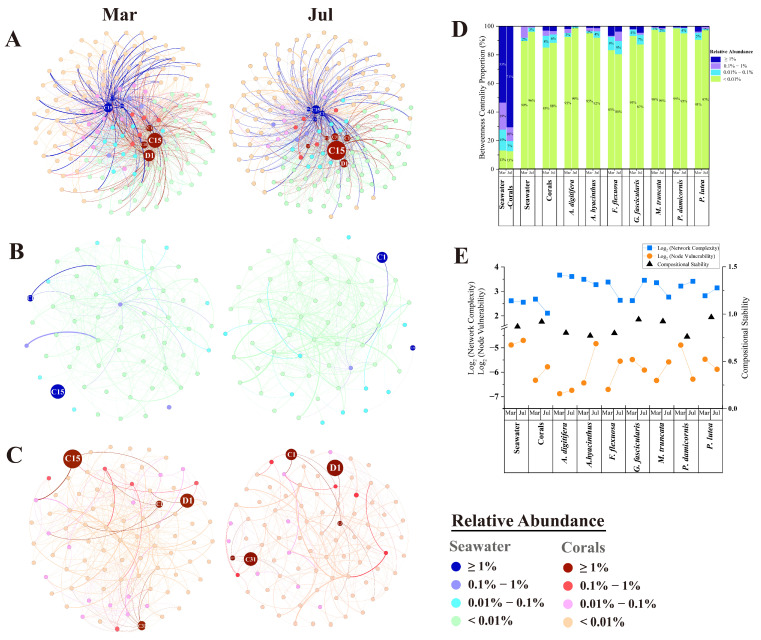
Free-living and in hospite Symbiodiniaceae networks in seawater and corals. (**A**) The cooccurrence network interaction analysis revealing among free-living and symbiotic Symbiodiniaceae genotypes. (**B**) The cooccurrence network based on Symbiodiniaceae genotypes within seawater. (**C**) The cooccurrence network of the endosymbiotic Symbiodiniaceae community within corals. Each node represents a Symbiodiniaceae genotype. Node size is proportional to the relative abundance of genotypes, and color indicates the range of the relative abundance, summarizing different levels of the relative abundance groups. Edge width is equivalent to the edge betweenness value. (**D**) The betweenness centrality distribution of the co-occurrence network based on Symbiodiniaceae communities within seawater, corals, and various coral species. (**E**) The network complexity, vulnerability, and compositional stability of the Symbiodiniaceae community in seawater, corals, and various coral species. The values of the network complexity and vulnerability were measured by the logarithmic ratio (logarithm to the base 2).

Using co-occurrence network analysis, we examined genotype associations in Symbiodiniaceae communities between seawater and corals (Figure 3A). The free-living and symbiotic Symbiodiniaceae network (FSSN) highlighted that Symbiodiniaceae abundant and intermediate types functioned as central hubs with higher connectivity. Compartmental segregation was evident between abundant and rare types within FSSN, with a negative co-occurrence relationship observed between free-living and symbiotic Symbiodiniaceae communities (Figure S7). In free-living networks (FSN), abundant genotypes occupied peripheral positions with low connectivity (Figure 3B). Contrarily, rare types formed central hubs with more associations in both free-living and symbiotic Symbiodiniaceae networks (SSNs), as observed across seven coral species (Figure 3C and Figure S8). Betweenness centrality analysis confirmed that abundant and intermediate genotypes exhibited higher connectivity, while rare genotypes served as critical hubs significantly influencing community interactions during both March and July (Figure 3D, Table S9). Topological properties (complexity, node vulnerability, and compositional stability) were compared across networks (Figure 3E). Complexity reflects the diversity and interconnectedness of Symbiodiniaceae genotypes and may indicate a functionally diverse and ecologically resilient community. Vulnerability represents the network’s sensitivity to node loss, highlighting the reliance on key genotypes and the potential risk of community collapse under environmental stress. Stability describes the capacity of the community to maintain its structure and interactions over time, serving as an indicator of enhanced resilience to environmental fluctuations. Network complexity declined significantly in July relative to March for both FSN and most SSNs, accompanied by increased vulnerability. This trend was consistent in the SSNs of *A. digitifera*, *A. hyacinthus*, *F. flexuosa*, and *Montipora truncate*. Contrastingly, *G. fascicularis*, *P. damicornis*, and *P. lutea* exhibited elevated complexity and reduced vulnerability in July. SSNs demonstrated greater compositional stability than FSN (Table S10). Among coral species, *P. lutea* and *G. fascicularis* hosted the most stable SSNs, while *A. hyacinthus* and *P. damicornis* displayed the lowest stability.

### 3.4. Correlations of Symbiodiniaceae Community with Environmental Factors

**Figure 4:**
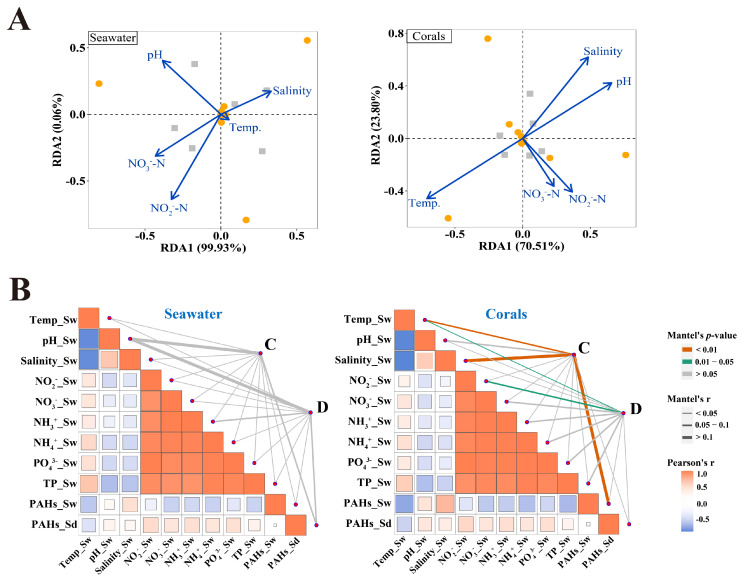
Environmental factors of Symbiodiniaceae community structure. (**A**) Relationships between the Symbiodiniaceae community and environmental factors determined by redundancy analysis (RDA). The gray and orange dots respectively represent Symbiodiniaceae communities in March and July. (**B**) Pairwise comparisons of environmental factors are shown with a color gradient denoting Spearman’s correlation coefficient. The letters C and D represent *Cladocopium* and *Durusdinium*, respectively. Sw and Sd, respectively, represent seawater and sediment. Community compositions of *Cladocopium* and *Durusdinium* were related to each environmental factor by Mantel tests. Line width corresponds to Mantel’s r statistic for the corresponding distance correlations, and line color denotes the statistical significance based on 9999 permutations.

RDA was performed following DCA to assess environmental drivers of Symbiodiniaceae community composition (Tables S11 and S12). The RDA revealed that the Symbiodiniaceae communities in seawater and corals were significantly affected by SST, pH and salinity, nitrite nitrogen (NO_2_^−^-N), and nitrate nitrogen (NO_3_^−^-N) on the whole (Figure 4A). Among these factors, SST had the strongest influence on the Symbiodiniaceae community in corals. However, *Cladocopium* and *Durusdinium* in seawater showed no significant correlations with environmental parameters (Figure 4B). Coral endosymbiotic *Cladocopium* and *Durusdinium* exhibited noticeable correlations (*p* < 0.05) with SST. Additionally, *Cladocopium* was markedly correlated with salinity, ∑PAHs of seawater, while *Durusdinium* correlated with NO_2_^−^-N. Genotype-level analysis displayed predominantly *Cladocopium* genotypes in corals showing (1) significant positive correlations (*p* < 0.05) with SST (Figure S9), and (2) significant negative correlations (*p* < 0.05) with pH, salinity, NO_2_^−^-N, NH_3_^+^-N, ammonia nitrogen (NH_4_^+^-N), phosphate (PO_4_^3−^-P), and ∑PAHs.

## 4. Discussion

### 4.1. Adaptability Regulation of Symbiodiniaceae Community Between Cold and Warm Months

*Durusdinium* is recognized as a stress-tolerant genus, particularly under temperature extremes, pollution, and disturbance stress [77,78,79,80] For example, *Durusdinium* can establish stable symbioses with host corals both in environments experiencing temperatures up to 30 °C or routinely below 20 °C [81,82] and under conditions characterized by high rates of temperature increase and Degree Heating Weeks (DHW) [83]; coral with a high abundance of *Durusdinium* was discovered in a severe anthropogenic disturbance region [29]. Typically, the optimal survival temperature range of scleractinian corals is 23–29 °C [84,85,86]. Many Symbiodiniaceae species show optimal growth and photosynthetic efficiency within the range of 25–30 °C [87]. Sustained cold temperatures from January to March (Figure 1A) likely reduced the growth rate of coral endosymbiotic Symbiodiniaceae. Noteworthy, the organic pollution and human disturbance in March during the peak tourist season were obviously higher than in July during the off-season on the whole (Table S11). These possibly induced pollution stress and severe anthropogenic disturbance, evidenced by high *Durusdinium* abundance and reduced *Cladocopium*/*Durusdinium* ratios (Figure S4 and Figure 1B). This likely inhibited *Cladocopium* growth in most hosts or caused the expulsion of these in hospite Symbiodiniaceae into the surrounding seawater [88], thereby resulting in a proportional increase in Durusdinium. This is possibly one of the contributing factors to the reduced diversity of in hospite Symbiodiniaceae observed during the colder months (Figure S3B). In contrast, while the evenness of Symbiodiniaceae in seawater was higher during the cold month, the richness did not differ significantly between the two sampling periods (Figure S3A). This suggests that free-living Symbiodiniaceae communities are less impacted by adverse environmental conditions compared to their symbiotic counterparts. The ambient Symbiodiniaceae community has higher diversity than the in hospite Symbiodiniaceae, but with shared dominant genotypes. Partial genotype overlap between coral and seawater (Figure 1D) and low distributional similarity (Figure 1E) indicate constrained Symbiodiniaceae exchange. These results indicated that even though the transfer of Symbiodiniaceae genotypes between coral and seawater was limited [89,90], there was still some movement between free-living and symbiotic Symbiodiniaceae. The relatively high abundance of *Durusdinium* genotypes in corals was found in cold months (Figure S4 and Table S3), and *Durusdinium* dominated in *F. flexuosa*, *G. fascicularis*, and *P. damicornis* (Figure 1C), enhancing these hosts’ tolerance responses to environmental stress [91,92]. Collectively, this study suggests that an increase in *Durusdinium*, or a shift toward *Durusdinium*-dominance, enhances the host’s tolerance and adaptability to extreme environmental fluctuations compared to conspecifics dominated by *Cladocopium*. In response to adverse environmental stressors, symbiont shuffling appears to serve as an adaptive regulation to enhance the in hospite symbiont survivability [79].

Following the cold season dynamics, we observed contrasting patterns during the warm months (Figure 1B). Symbiont shuffled from *Durusdinium* to *Cladocopium* in these coral species, indicating coral possibly recovered from environmental stress. Numerous studies have demonstrated that environmental stressors, such as pollution or extreme temperature [41,79], trigger symbiont shuffling toward increased *Durusdinium*, even culminating in *Durusdinium*-dominance in coral’s Symbiodiniaceae communities [32,33,93]. Although *Durusdinium* enrichment may enhance host stress resilience [94], this trait came at the expense of low nutrient utilization efficiency in unstressed environments [95]. It was documented to exhibit reduced calcification [96], growth [35], nitrate assimilation [97], and carbon translocation in corals under non-stressful conditions [5]. When disadvantageous environmental conditions subside, host corals might restore *Cladocopium* abundance or shift to *Cladocopium*-dominance [41,94], aiming to improve symbiosis survival efficiency [98]. Despite greater stress sensitivity, *Cladocopium* exhibited higher photosynthetic efficiency and translocated more fixed carbon and nitrogen compared with *Durusdinium* under no stress [99]. *Cladocopium*-dominated corals were observed to have higher growth rates and autotrophic nutritional capacity than *Durusdinium*-dominated corals [35,100,101,102]. For example, the symbioses of coral and *Cladocopium* were extensively observed in the absence of enduring long-term stress or under consistently favorable environmental conditions, where they exhibit higher autotrophic efficiency [102,103]. These findings suggest that *Cladocopium*-dominated corals exhibit greater nutrient acquisition efficiency (e.g., carbon and nitrogen) through symbionts [5], resulting in accelerated growth rates [97], and highlight the competitive advantage of *Cladocopium* in supplying energy and nutrients to the host corals under long-term suitable environmental conditions. Under persistently low-stress reef conditions, the expulsion of *Cladocopium* from the coral host perhaps decreased, and its growth within the host was restored, thereby reducing the relative abundance of *Durusdinium* [41]. *Cladocopium*-dominated corals showed the greater survival advantage in suitable/unstressed conditions [5]. Therefore, the symbiont shuffling from *Durusdinium* to *Cladocopium* dominance represents a regulatory adaptation to maximize their symbiotic survival advantage in less stressful environments.

Sustained elevated temperatures occurred from June to September (Figure 1A), and SST at Luhuitou and Phoenix Island reached 30.4 °C and 30.6 °C during July (Table S11). Declining *Cladocopium*/*Durusdinium* ratios were observed in *A. digitifera* and *P. lutea* at Luhuitou and *G. fascicularis* and *P. lutea* at Phoenix Island (Figure 1B), with *G. fascicularis* at Phoenix Island shifting to *Durusdinium* dominance. These indicated the presence of heat stress for corals at the two sites in warm months. According to the above discussion, increasing the abundance of *Durusdinium* or shifting towards *Durusdinium*-dominance could improve the thermotolerance of the coral host [94,96]. In addition, *F. flexuosa*, *G. fascicularis*, and *P. damicornis* might have rapid adaptive regulation of endosymbiotic Symbiodiniaceae in response to cold and heat stress, especially *G. fascicularis*. Genotype-level shuffling was observed between dominant *Cladocopium* genotypes (C15, C31, C1) and *Durusdinium* (D1) (Figure S6). These suggested that temperature adaptability regulation of the Symbiodiniaceae community could be achieved by the shuffling between stress-tolerant *Durusdinium* and sensitive *Cladocopium* genotypes. It has been previously reported that the thermotolerance hierarchy was D1 > C15 > C31 > C1 [104]. Therefore, D1 might have the strongest cold and heat tolerance among these genotypes, combined with the results of this study; similarly, C15 could have lower thermal sensitivity than C31 and C1. Symbiodiniaceae genetic diversity increased during warm months, primarily driven by temporal turnover of rare genotypes within the *Cladocopium* and *Durusdinium* genera (Figure S3A–C and Figure 2). Rare genotypes may serve as a genetic reservoir, enhancing holobiont resilience under fluctuating environmental conditions [105]. Temporal turnover has marked effects on the structure and functioning of ecological communities and can be rapid even while the number of species remains relatively unchanged [106]. Proliferation of rare Symbiodiniaceae genotypes typically occurs during and post-bleaching events, thermal stress, or PAH stress, concomitant with Symbiodiniaceae shuffling [45,92,107,108]. As a result, rare *Durusdinium* genotypes might confer adaptive potential for future stressors, whereas rare *Cladocopium* genotypes could enhance survival advantage under suitable environments [97,109].

### 4.2. Seasonal Changes in Symbiodiniaceae Interaction and Community Stability

Building on the role of rare genotypes, we next examined how network properties reflect coral species’ resilience. The analysis of the interaction between free-living and symbiotic Symbiodiniaceae (Figure 3A and Figure S7, Table S9) and the discussion in Section 4.1 implied that free-living Symbiodiniaceae in seawater were mainly influenced by coral expulsion of abundant and intermediate types [110]. Intriguingly, rare types might play a more significant role in genotypic interactions within Symbiodiniaceae communities in both cold and warm months than abundant or intermediate types (Figure 3B,C and Figure S8 and Table S9), which appeared to be unaffected by temperature changes. This aligned with the prediction of the network theoretic modeling approach to coral-Symbiodiniaceae associations, symbolizing that low-abundance Symbiodiniaceae could greatly enhance the stability of coral-Symbiodiniaceae symbioses [24]. Furthermore, rare genotypes might enhance the potential resilience of the coral host by replacing dominant genotypes lost during environmental stress [48]. Functionally rare genotypes may replace dominant types to preserve key ecological processes in host corals under environmental fluctuations [111,112]. Rare Symbiodiniaceae genotypes may play a critical role in coral recovery following environmental stress [94]. Overall, our findings suggest that Symbiodiniaceae interactions serve as indicators of community stability and may reflect the thermal tolerance or sensitivity of coral hosts under environmental stress.

Numerous studies on coral-symbiont networks have shown that the interactions within these networks are closely linked to the health and resilience of corals [30,105,113]. High stability within coral–Symbiodiniaceae networks is often associated with highly connected, generalist symbionts that exhibit greater environmental tolerance. The coral hosts were the weaker partner in the coral-symbiont network when the coral holobionts were under environmental stress [114]. Dominant Symbiodiniaceae genotypes may influence interactions between symbiotic algae and associated fungal communities [21]. Collectively, these findings suggested that Symbiodiniaceae interactions had a potentially dominant impact on associations within coral symbionts. The change results of SSN complexity and vulnerability in warm months (Figure 3E) indicated that endosymbiotic Symbiodiniaceae interactions in most coral species became weakened, potentially enhancing the fragility of Symbiodiniaceae communities and the sensitivity of coral symbionts. Microorganisms form a complex network of interactions closely related to community stability [115]. It has been reported that the stability of microorganism networks increases with network complexity [116]. Elevated SST in warm months may disrupt genotype interactions, potentially affecting holobiont stability. Noteworthy, the changes in SSN complexity and vulnerability in *G. fascicularis*, *P. damicornis*, and *P. lutea* (Figure 3E) portended that Symbiodiniaceae interactions in these three coral species appeared to be more stable in the warm month compared to the cold month. Furthermore, the stabilities of Symbiodiniaceae networks (Table S10) denoted that *G. fascicularis* and *P. lutea* with stabilized Symbiodiniaceae communities were most tolerant in the seven coral species under reef temperature fluctuation from the cold to the warm months, while *A. digitifera* and *P. damicornis* with fragile Symbiodiniaceae communities were more sensitive. This observation aligns with previous studies indicating that *Galaxea* spp. and *Porites* spp. are stress-tolerant with relatively stronger temperature tolerance, while *Acropora* spp. and *Pocillopora* are stress-sensitive [117,118,119]. Acroporidae and Pocilloporidae showed greater susceptibility compared to *Galaxea* and *Porites* during El Niño-induced bleaching events [120,121], also reflecting the temperature sensitivity of the former and tolerance of the latter. Consistently, *Acropora* spp. and *P. damicornis* demonstrated greater bleaching sensitivity and susceptibility, whereas *P. lutea* showed better endurance to both heat and cold stress [122,123,124]. To sum up, we considered that Symbiodiniaceae interactions could reflect the stabilization and fragility levels of the Symbiodiniaceae community and the temperature tolerance and sensitivity levels of coral symbionts.

### 4.3. Effects of Environmental Factors on Free-Living and In Hospite Symbiodiniaceae Community

Among all environmental factors examined, temperature exerted the most significant influence on coral-symbiotic Symbiodiniaceae community dynamics (Figure 4A,B). Simultaneously, the symbiotic Symbiodiniaceae community was significantly affected by pH, salinity, nutrient levels, and organic contaminants. Normally, the optimal pH for scleractinian corals ranged from 8.0 to 8.5 [84,85], and the ideal salinity for coral symbiotic *Cladocopium* is 28–32‰ [125]. Coral universal tolerance thresholds span salinity 28.7–40.4‰, nitrate ≤ 4.51 μmol·L^−1^, and phosphate ≤ 0.63 μmol·L^−1^ in general, respectively [126]. Although seawater salinity (31–37‰) and pH (7.9–8.1) remained within general coral tolerance limits (Table S11), nitrogen (0.1–22 μmol·L^−1^) and phosphorus (0.3–1.4 μmol·L^−1^) levels exceeded the general coral tolerance levels. Nitrate/nitrite/phosphate eutrophication drove *Cladocopium* replacement by other genera and reduced the number of *Cladocopium*-dominated corals [127]. Elevated PAH concentrations in cold-month seawater/sediments correlated with peak tourism and dry conditions, predominantly originating from anthropogenic sources [128]. These findings indicate that corals during the cold season were exposed to increased anthropogenic disturbance and organic pollution. Anthropogenically disturbed reefs showed reduced *Cladocopium* and enhanced *Durusdinium* abundance [29], similar to Symbiodiniaceae community changes under seawater PAH stress [92,129]. It was reported that corals in minimally disturbed coral reefs were typically dominated by *Cladocopium*, while corals from areas exposed to high anthropogenic pressure were dominated by *Durusdinium* [130]. Interestingly, the genotypes most strongly correlated with the ∑PAH concentrations in sediments belong to *Cladocopium*, possibly indicating the potential impact of sediment ∑PAHs concentrations on Symbiodiniaceae community changes in corals. Sediment resuspension plays a critical role in mediating seawater contamination and exacerbating coral symbiont stress [131]. PAHs often accumulate in benthic sediments due to their low solubility and strong affinity for particulate matter [132]. Physical disturbances (such as wave action and storms) and anthropogenic activities (coastal tourism and dredging) can resuspend contaminated sediments into the water column, thereby increasing the concentration of PAHs in the surrounding seawater [133]. Once released, these pollutants pose significant toxicity risks to coral symbionts. Studies have demonstrated that elevated PAH levels in seawater impair the photosystem function of symbiotic Symbiodiniaceae [128], trigger oxidative stress responses [134]. Concurrently, PAH stress can lead to symbiont shuffling, which is typically characterized by a decline in *Cladocopium*, an increase in the more stress-tolerant *Durusdinium* [92], and the emergence of rare Symbiodiniaceae genotypes [108]. These suggest that sediment-derived PAHs, through resuspension-driven seawater contamination, may act as a key stressor undermining coral holobiont stability and resilience. However, coral-symbiotic Symbiodiniaceae communities may be simultaneously influenced by multiple interacting environmental stressors [135]. These changes in the Symbiodiniaceae community could be primarily driven by temperature interacting with adverse environmental factors, including anthropogenic disturbance, elevated salinity, and environmental pollution, that might disadvantage *Cladocopium* relative to *Durusdinium*. Therefore, temperature-driven adaptability regulation of the symbiotic Symbiodiniaceae community was likely to be aggravated by multiple adverse environmental factors.

By contrast, ambient seawater Symbiodiniaceae (*Cladocopium* and *Durusdinium*) inhabit a very different ecological niche than those inside corals. In our sites, a portion of free-living Symbiodiniaceae cells is likely derived from corals via routine expulsion or stress-related release. [136]. Once released into the water column, these cells are widely dispersed and diluted, so they form a mixed, transient population. Free-living Symbiodiniaceae communities are thus dominated by stochastic dispersal and random loss rather than by local environmental filtering. [88]. For example, strong water movement and grazing can rapidly homogenize planktonic assemblages, producing unpredictable fluctuations that do not track steady environmental gradients. Consequently, ambient *Cladocopium* and *Durusdinium* abundances may vary independently of measured parameters. Given these factors (host-derived input, high dispersal, and temporal stochasticity), the absence of a strong correlation between seawater symbionts and environmental variables is ecologically reasonable.

### 4.4. Temperature-Driven Variations in In Hospite Symbiodiniaceae Community

Cold temperature reduced Symbiodiniaceae photochemical efficiency and *Cladocopium* abundance while increasing *Durusdinium* dominance [39,137]. *Durusdinium*-dominated corals displayed a 1–2 °C lower cold stress threshold compared to *Cladocopium*-dominated corals [138], and yet they experienced the post-stress shuffling towards *Cladocopium* upon recovery [41]. Sustained cold temperatures from January to March (Figure 1A, Table S11) remained within the optimal coral growth range (23–29 °C). Nonetheless, corals in cold months simultaneously faced salinity fluctuations, organic pollution, nutrient enrichment, and anthropogenic disturbance (Section 4.3). Cold-driven shifts toward *Durusdinium* dominance were likely exacerbated by these additional stressors. During warm months, temperature and other environmental factors returned to levels within the coral adaptation range (Table S11), facilitating *Cladocopium* recovery in most corals (Figure 1C). It was a reason that genotypes most positively correlated with SST belonged to the genus *Cladocopium* on the whole but negatively correlated with salinity, nitrogen and phosphorus nutrients, and PAHs (Figure S9). Although SST reached 30.4 °C at Luhuitou (Table S11), *Cladocopium*-dominated corals did not exhibit a shift toward *Durusdinium* dominance. However, it is worth noting that SST and salinity at Phoenix Island rose by 30.6 °C and 34‰, and the concentrations of nitrate and phosphate in seawater far exceeded the tolerance levels of corals in warm months. Elevated salinity and enriched nutrients possibly enhanced the high temperature-driven shuffle from *Cladocopium*-dominated *G. fascicularis* to *Durusdinium*-dominance, and *Cladocopium*-*Durusdinium* mixed dominated in *P. damicornis*. *Cladocopium*-dominated corals exhibited greater photochemical damage and a quicker increase in the proportion of *Durusdinium* in the Symbiodiniaceae community under thermal stress [35]. Even if it might occur, shuffling to tolerate *Durusdinium* dominance [139], accompanied by the restructuring of Symbiodiniaceae genotypes [109], *Durusdinium*-dominated corals exhibited higher resistance to heat stress with mild physiological stress and higher carbon assimilation and nutrient transformation than *Cladocopium*-dominance [26,39,104,140]. *Durusdinium* enrichment might constitute a defense mechanism against high temperatures [141]. For instance, *Platygyra verweyi* shifted from *Cladocopium*- (C3 and Ccc) to thermally tolerant *Durusdinium*-dominance (D1a) following thermal stress events, elevating thermal tolerance by 1.0–1.5 °C [82]. Additionally, nutrient enrichment would disrupt the nitrogen balance between Symbiodiniaceae and the coral host [142], exacerbating coral response to thermal stress [143]. Noteworthy, Phoenix Island’s elevated PAH levels might lead to physical and oxidative stress [58,134,144,145], more sensitizing corals to thermal stress. Temperature acted as the predominant factor affecting the in hospite Symbiodiniaceae community in seasonal variation, whereas the concurrence of salinity fluctuations, organic pollution, and severe anthropogenic disturbance was the driving factor in cold months, causing in hospite Symbiodiniaceae shuffling and temporal turnover of rare genotypes. Furthermore, seasonal variations in the Symbiodiniaceae community were concurrently influenced by mutual environmental factors, mainly including pollution and anthropogenic disturbance, possibly exacerbating the temperature sensitivity of coral hosts.

**Figure 5:**
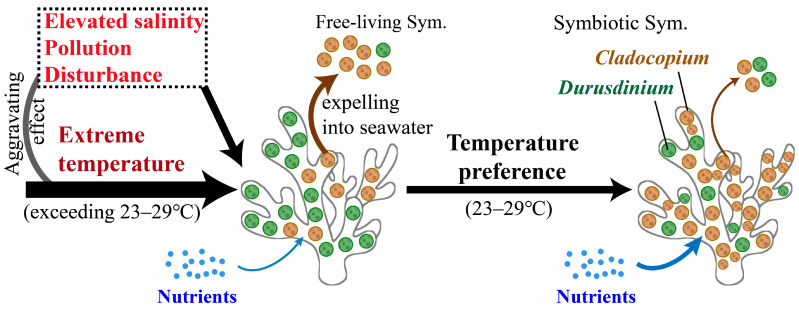
Conceptual model illustrating temperature-driven adaptability regulation of in hospite Symbiodiniaceae community in coral reefs.

Based on the present and previous results, we propose a conceptual model illustrating the temperature-driven adaptability regulation of the symbiotic Symbiodiniaceae community in coral reefs (Figure 5). Both cold and hot temperatures can drive Symbiodiniaceae to shift to stress-tolerant *Durusdinium*, enhancing host resilience but reducing nitrate assimilation, carbon translocation, and growth [5,35,97]. Furthermore, the temperature-driven adaptability regulation of in hospite Symbiodiniaceae in coral reefs could be through Symbiodiniaceae shuffling and rare genotype turnover. The phenomena might be aggravated by mutual factors, including elevated salinity, organic contamination, nutrient enrichment, and anthropogenic disturbance. Also, these mutual factors directly induced the response of the in hospite Symbiodiniaceae community. *Cladocopium* dominance optimizes coral nutritional efficiency and growth under favorable environmental conditions [99], which enhances the competitive advantage of the coral host. However, host-specific tolerance thresholds for environmental factors further determine Symbiodiniaceae adaptive regulation capacity [33,130].

## 5. Conclusions

This study investigated the dynamics of in hospite and free-living Symbiodiniaceae communities in coral reefs between cold and warm months. We aimed to explore the adaptability regulation of the coral endosymbiotic Symbiodiniaceae community in seasonal variation. We found that seasonal variation occurred in the in hospite Symbiodiniaceae community around Hainan Island, China. Our results demonstrate that adaptability regulation in coral-symbiotic Symbiodiniaceae communities occurs through symbiont shuffling and turnover of rare genotypes, thereby enhancing the acclimatization ability of coral hosts. The flexible shuffling between *Durusdinium* and *Cladocopium* genotypes could improve the tolerance and adaptability of the coral holobiont to environmental changes. *Durusdinium*-dominated corals exhibited greater environmental adaptability and thermal tolerance, albeit with reduced nutrient utilization efficiency. *Cladocopium*-dominated corals with highly efficient nutritional efficiency had the greater survival advantage in suitable/unstressed conditions. Symbiodiniaceae shuffling from *Durusdinium* to *Cladocopium* dominance represented a regulatory adaptation to maximize their symbiotic survival advantage in less stressful environments. The temporal turnover of rare genotypes might provide increased options for coping with future stress and enhance survival advantages under suitable environmental conditions. Rare genotypes acted as central hubs within Symbiodiniaceae networks, remaining stable across temperature fluctuations. This suggested their crucial role in maintaining Symbiodiniaceae community structure stability and highlighted their potential importance for coral holobiont resilience. Thermal temperature increased Symbiodiniaceae community fragility and coral potential sensitivity. *G. fascicularis* and *P. lutea* maintained stabilized Symbiodiniaceae communities with greater temperature tolerance compared to *A. digitifera* and *P. damicornis*. Consequently, we suggested that Symbiodiniaceae interaction dynamics could signal the community stabilization and fragility, as well as the environmental tolerance and sensitivity of coral hosts. Additionally, temperature-driven Symbiodiniaceae community variations were concurrently influenced by other environmental factors, potentially exacerbating the temperature sensitivity of coral hosts. Temperature-driven symbiont shuffling and rare genotype turnover may be exacerbated by concurrent stressors, including elevated salinity, organic contamination, nutrient enrichment, and anthropogenic disturbance. Overall, these findings enhance our understanding of how the Symbiodiniaceae community influences coral host acclimatization and tolerance through its regulation of environmental adaptability. The study may provide a foundation for assessing the resistance of corals to environmental stress in the context of global climate change.

## Data Availability

The data presented in this study are openly available in the NCBI Sequence Read Archive Database with accession number PRJNA1148647.

## Supplementary Materials

The following supporting information can be downloaded at https://www.mdpi.com/article/10.3390/microorganisms13081958/s1, Figure S1: Coral bleaching alert levels in Hainan (A, data from NOAA: https://coralreefwatch.noaa.gov/, the access date is 3 May 2025) and the sampling sites in Sanya coral reefs, Hainan, China (B); Figure S2: Rarefaction curves and species accumulation curves of operational taxonomic unit (OTU) in all samples of seawater (A) and corals (B); Figure S3: Alpha diversity of Symbiodiniaceae community in seawater (B), corals (C), and various coral species (D); Figure S4: The relative abundance of the genus levels of Symbiodiniaceae within seawater and all corals in March and July; Figure S5: Circular maximum likelihood phylogenetic tree and relative abundance of genotypes. The tree was constructed with MEGA X using the maximum likelihood method with a bootstrap value of 500 and displayed using Interactive Tree of Life (iTOL). The relative abundances of genotypes were shown in the heatmap as measured by the logarithmic ratio (logarithm to the base 10). The letters L, PI, and WI represent Luhuitou, Phoenix Island, and Wuzhizhou Island, respectively; Figure S6: Symbiodiniaceae community (the top 20 genotypes listed in descending order of relative abundance) in various coral species; Figure S7: Significant co-occurrence and co-exclusion relationships among genotypes in Symbiodiniaceae community. The node size is proportional to the relative abundance of genotypes in the host corals. The edge width is equivalent to the edge betweenness values. The edge color denotes co-occurrence (green) and co-exclusive (red) relationships; Figure S8: The networks of Symbiodiniaceae in various coral species. The node size is proportional to the relative abundance of symbiont genotypes in the host corals. The edge width is equivalent to the edge betweenness values; Figure S9: Heat maps (DHCA analysis and Pearson correction analysis) for relationships between the environmental factors and the genotypes abundance in seawater (A) and corals (B) (*, *p* < 0.05; **, *p* < 0.01); Table S1: The mean month sea surface temperature (SST) in Hainan Island, China; Table S2: Description for sampling stations and coral samples in this study; Table S3: Summary for the genus level sequence number and relative abundance of Symbiodiniaceae in coral reefs in March and July; Table S4: Alpha diversity of Symbiodiniaceae in seawater and corals; Table S5: Summary for the genus levels relative abundance of Symbiodiniaceae in seawater and various coral species at the sampling sites; Table S6: Symbiodiniaceae genotypes with significant differences in the relative abundance between March and July; Table S7: Summary of the sequence number and relative abundance of Symbiodiniaceae genotypes in seawater and corals in March and July; Table S8: The various genotypes relative abundance of Symbiodiniaceae within seawater and coral species samples at the sampling sites; Table S9: The node betweenness centrality values of the network based on Symbiodiniaceae community in seawater, corals, and various coral species; Table S10: The network complexity and stability of Symbiodiniaceae in seawater, corals, and various coral species; Table S11: Spatial and temporal distribution of the environmental factors in Sanya coral reefs, Hainan, China; Table S12: The decision curve analysis results for Symbiodiniaceae composition in seawater and corals; Excel: The genotypes relative abundance of Symbiodiaceae among seawater and coral species samples at the sampling sites. Refs. [59,60,128,146] can be found in Supplementary Materials.
