# Supplementary material for: Seasonal Variation in In Hospite but Not Free-Living, Symbiodiniaceae Communities Around Hainan Island, China
Source: Microorganisms. 2025 Aug 21;13(8):1958. doi: 10.3390/microorganisms13081958 (PMC12388015; doi:10.3390/microorganisms13081958)
Supplement: Supplementary file 1 [file microorganisms-13-01958-s001.zip › Supplementary Material 12_ YTH20250821.pdf]

Seasonal Variation in In Hospite but Not Free-Living, Symbiodiniaceae Communities Around Hainan Island, China

Tinghan Yang [1,2], Zhao Qi [3], Haihua Wang [4], Pengfei Zheng [5], Shuh-Ji Kao [2,*] and Xiaoping Diao [2,*]

[1] College of Ocean and Earth Sciences, Xiamen University, Xiamen 361102, China; yangtinghan@126.com
[2] State Key Laboratory of Marine Resource Utilization in South China Sea, Hainan University, Haikou 570228, China
[3] Department of Animal Ecology & Systematics, Justus Liebig University, 35392 Giessen, Germany; island517@126.com
[4] College of Life Sciences, Hainan Normal University, Haikou 571158, China; wanghaihua75@139.com
[5] Fourth Institute of Oceanography, Ministry of Natural Resources, Beihai 536000, China; zhengpengfei122@126.com
* Correspondence: sjkao@xmu.edu.cn (S.-J.K.); diaoxip@hainanu.edu.cn (X.D.)

Supporting Information

Supplementary Methods

1.1 Environmental factors determination

The filtered seawater and sediments were used for the determination of environmental parameters. Chemical and physical parameters of surface seawater were measured using the Chinese National Standard Methods (GB/T 12763.4-2007) [1] and a multi-function water quality analyzer (YSI, Professional Plus, USA), respectively. Sixteen polycyclic aromatic hydrocarbons (∑PAHs, the concentration of each polycyclic aromatic hydrocarbon was displayed in Yang, *et al.* [2]) in seawater (1L per sample) and sediments were collected through matrix solid-phase extraction and analyzed using gas chromatography of Agilent 7890 gas chromatograph coupled with a mass spectrometer of a 7000-mass selective detector (Agilent, Palo Alto, CA, USA). The procedural mean recoveries and the relative standard deviation for sixteen individual polycyclic aromatic hydrocarbons in seawater and sediments samples were validated (n = 5 seawater samples, 72.5%–109.7% recovery, and 5.9%–21.5% deviation. n = 6 seawater samples, 68.4%–112.9% recovery, and 51.3%–12.6% deviation). Specific procedures for detection, analysis, and recovery of analysts of chemical/physical parameters and ∑PAHs in corals and seawater samples were detailed in and Yang, *et al.* [3].

1.2 ITS2 rDNA amplification and purification

Each PCR reaction contained 9.25 µL Taq HotStart mix buffer (TaKaRa, Japan), 1 µM of each forward and reverse primer, 20 ng of total DNA, and DNAsefree water to a total volume of 50 µL. The PCR amplification was performed with the following program: 94 °C for 5 min, followed by 30 cycles of 95 °C for 30 s, 52 °C for 30 s, 72 °C for 60 s, with a final extension at 72 °C for 10min. All PCR products, including the negative kit and PCR controls, underwent electrophoresis with 1% agarose gel for screening. The PCR products were purified using TaKaRa MiniBEST Agarose Gel DNA Extraction Kit (TaKaRa, Japan).

Supplementary Figures

A

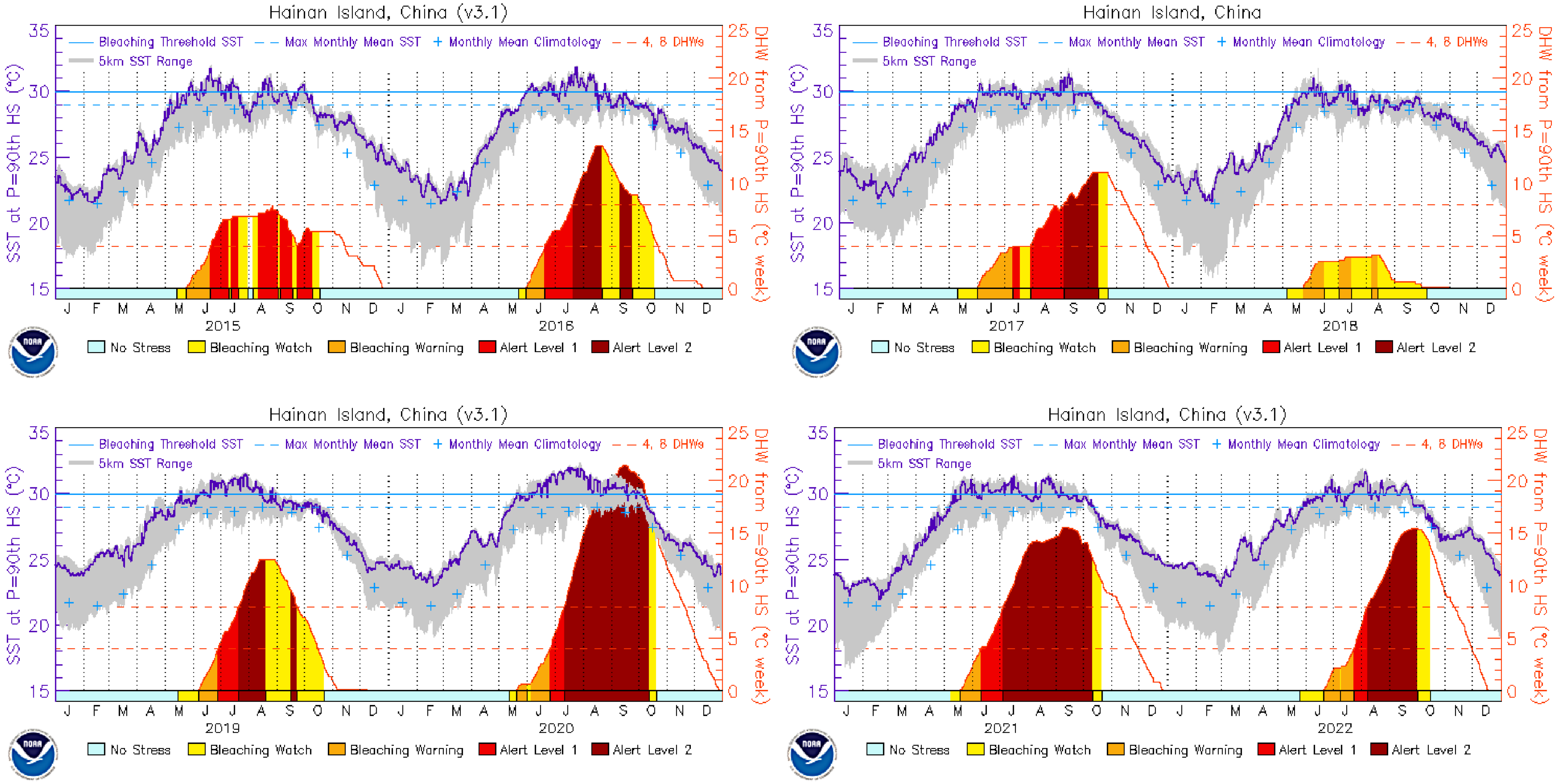

B

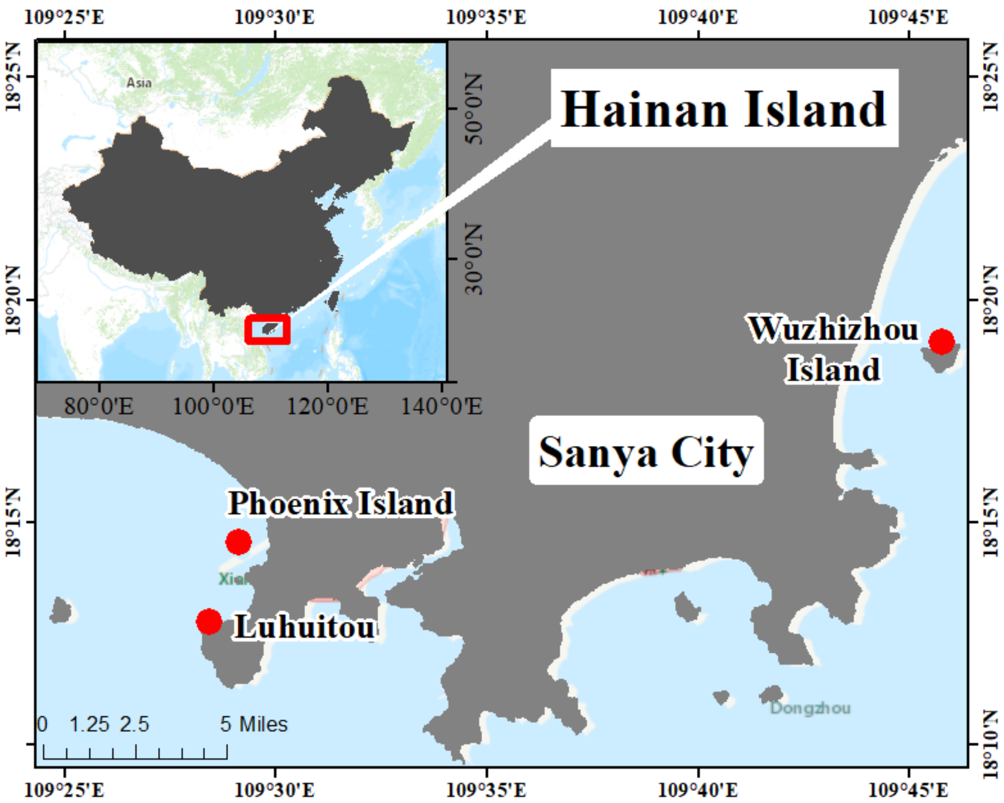

Fig. S1 Coral bleaching alert levels in Hainan（A, data from NOAA: https://coralreefwatch.noaa.gov/, the access date is 03 May 2025) and the sampling sites in Sanya coral reefs, Hainan, China (B).

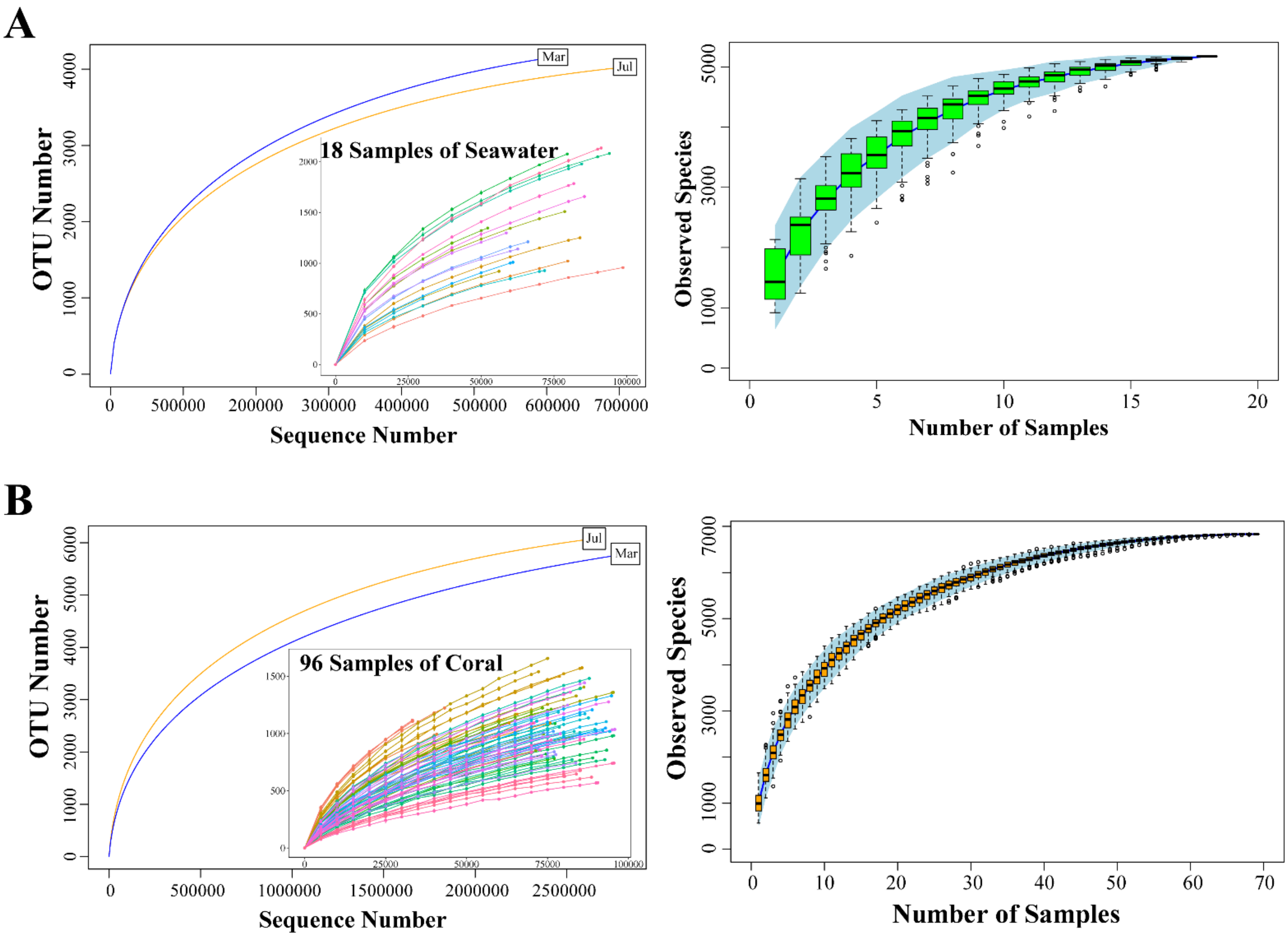

Fig. S2 Rarefaction curves and species accumulation curves of operational taxonomic unit (OTU) in all samples of seawater (A) and corals (B).

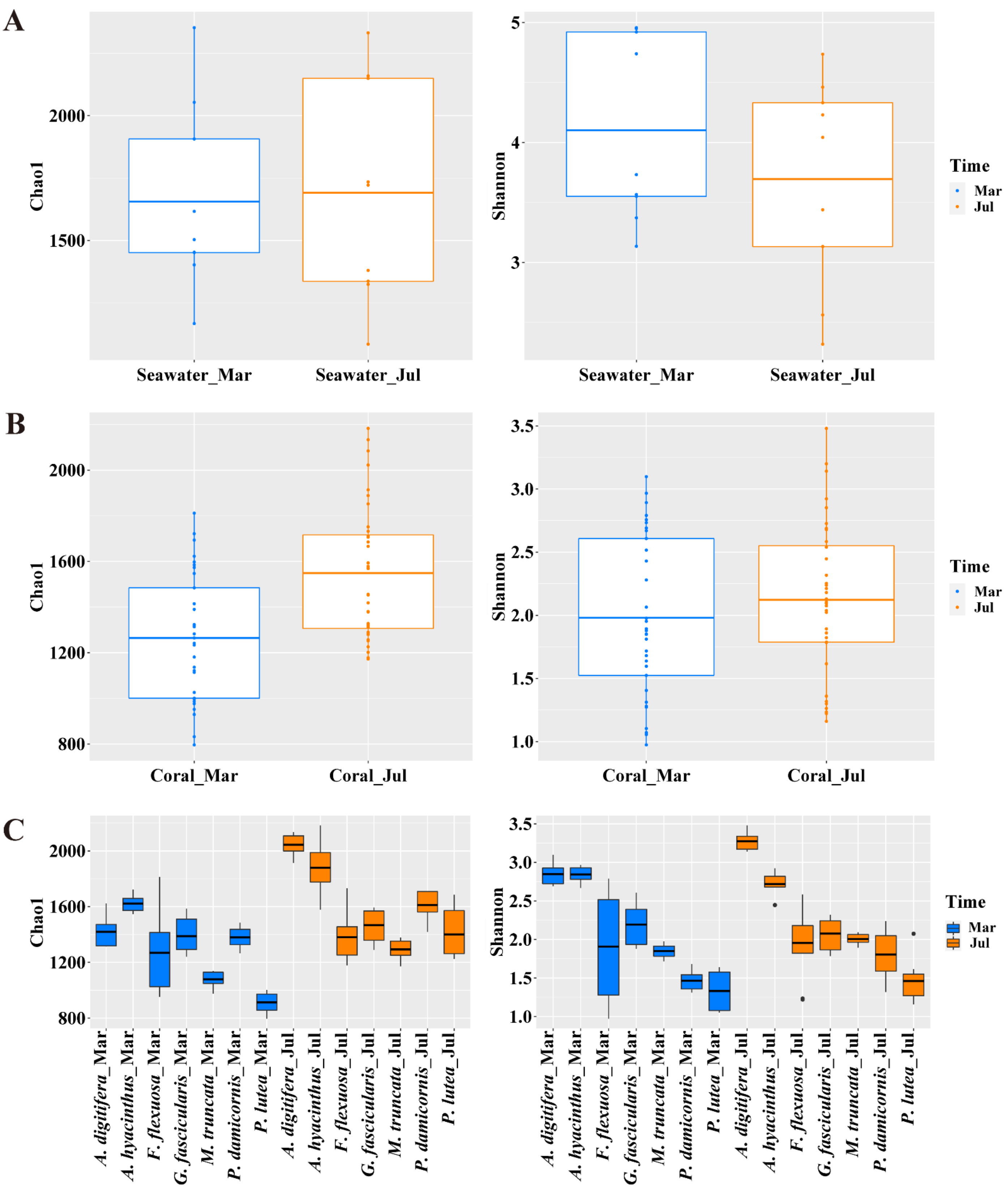

Fig. S3 Alpha diversity of Symbiodiniaceae community in seawater (B), corals (C), and various coral species (D).

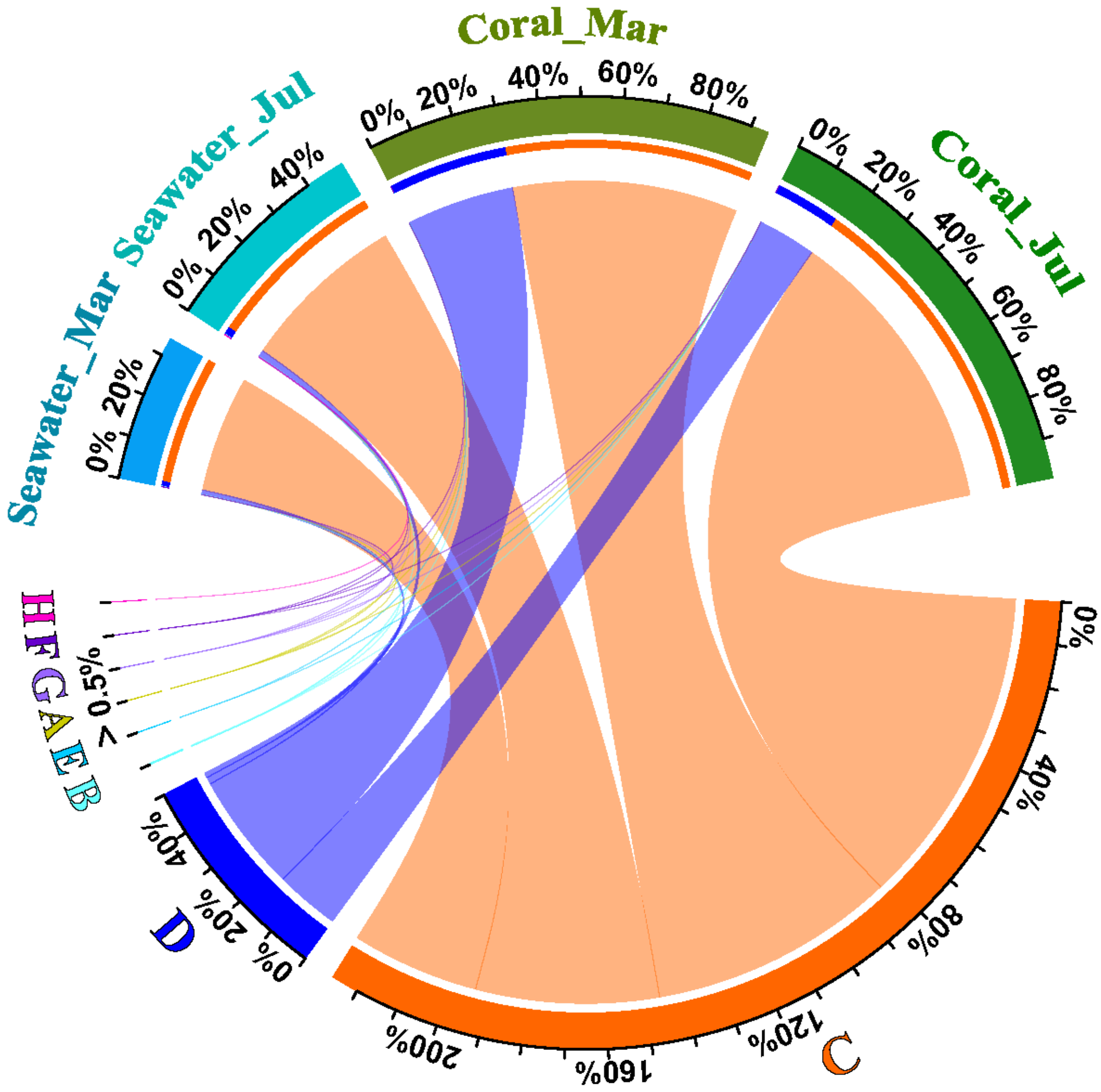

Fig. S4 The relative abundance of the genus levels of Symbiodiniaceae within seawater and all corals in March and July.

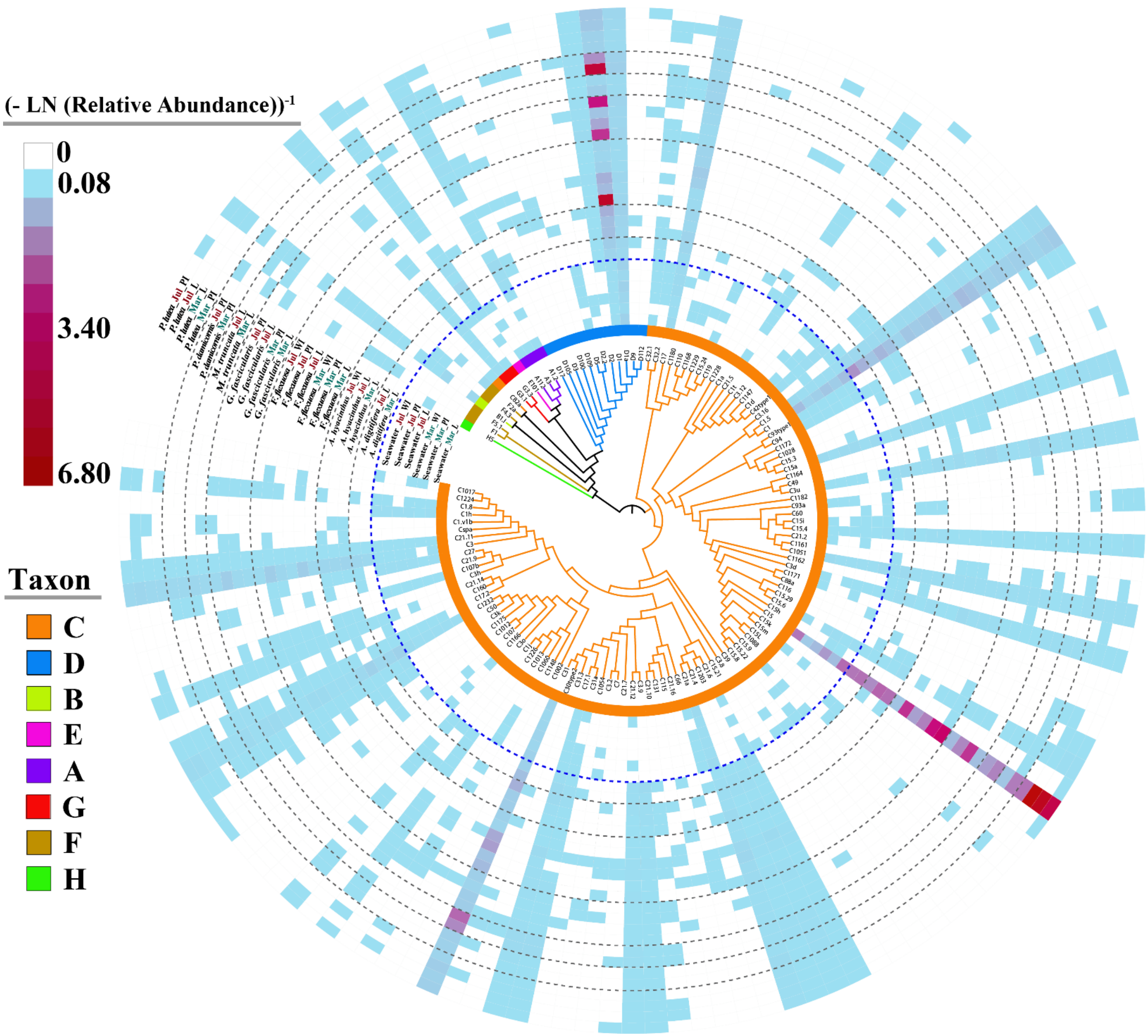

Fig. S5 Circular maximum likelihood phylogenetic tree and relative abundance of genotypes. The tree was constructed with MEGA X using the maximum likelihood method with a bootstrap value of 500 and displayed using Interactive Tree of Life (iTOL). The relative abundances of genotypes were shown in the heatmap as measured by the logarithmic ratio (logarithm to the base 10). The letters L, PI, and WI represent Luhuitou, Phoenix Island, and Wuzhizhou Island, respectively.

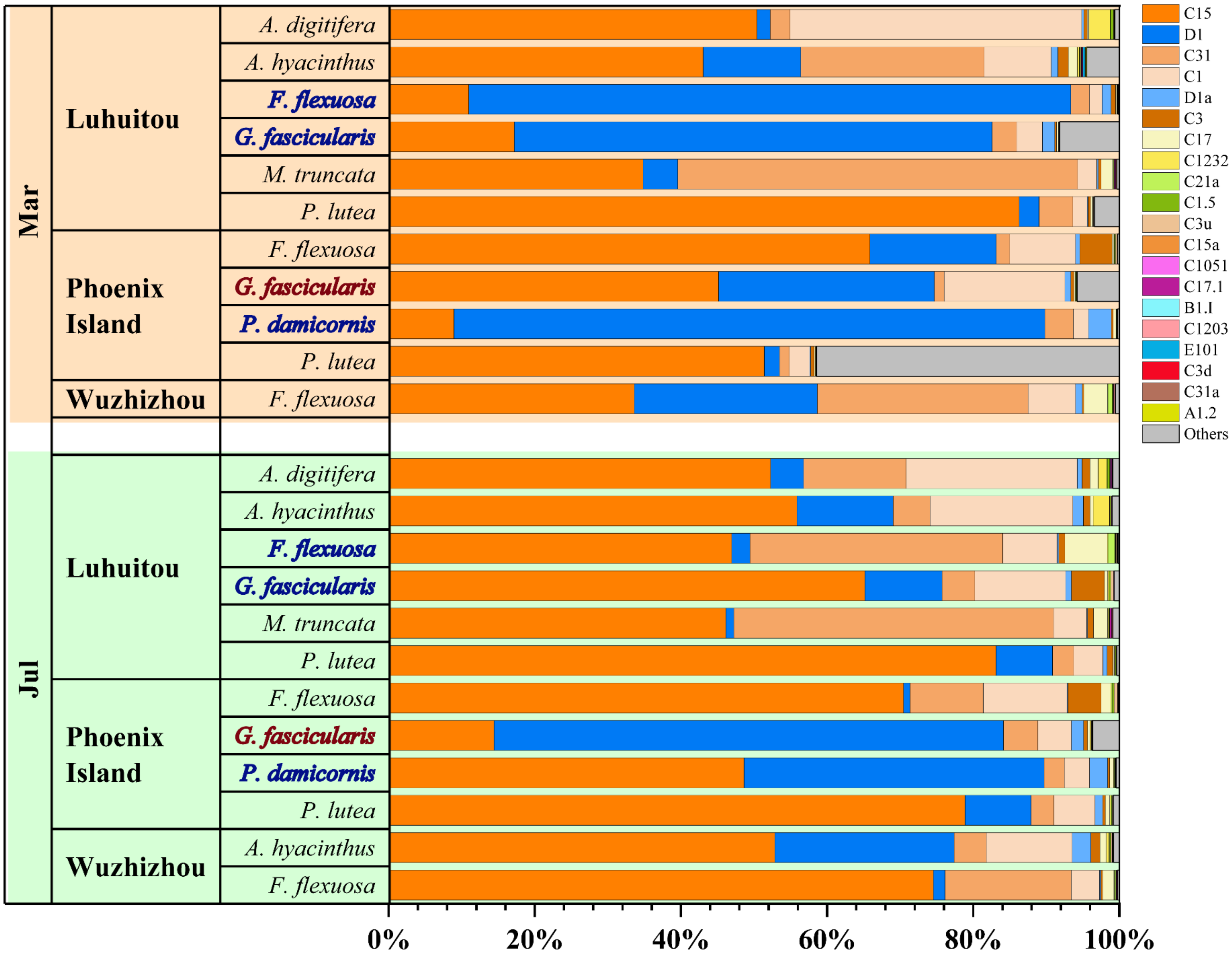

Fig. S6 Symbiodiniaceae community (the top 20 genotypes listed in descending order of relative abundance) in various coral species.

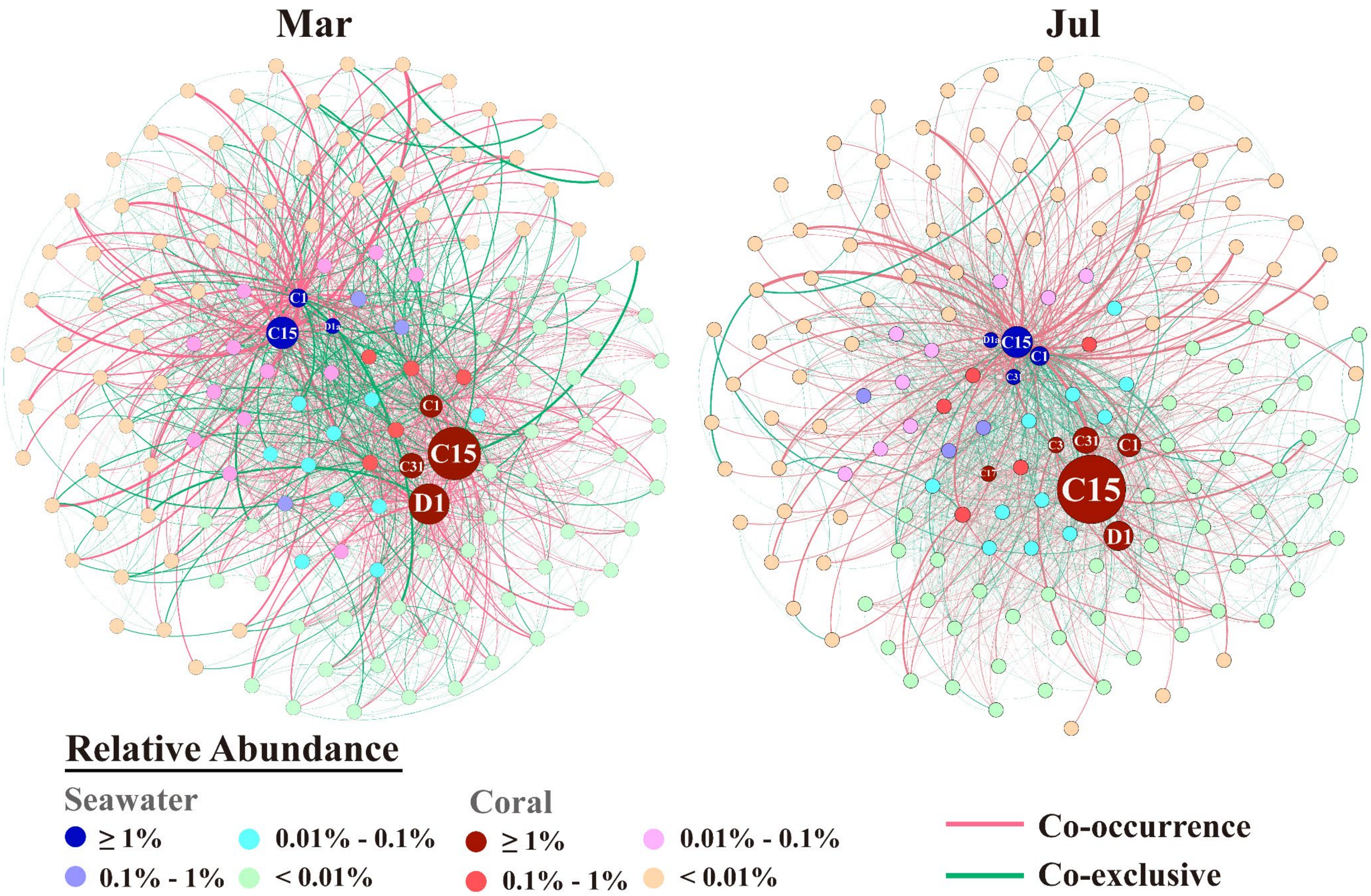

Fig. S7 Significant co-occurrence and co-exclusion relationships among genotypes in Symbiodiniaceae community. The node size is proportional to the relative abundance of genotypes in the host corals. The edge width is equivalent to the edge betweenness values. The edge color denotes co-occurrence (green) and co-exclusive (red) relationships.

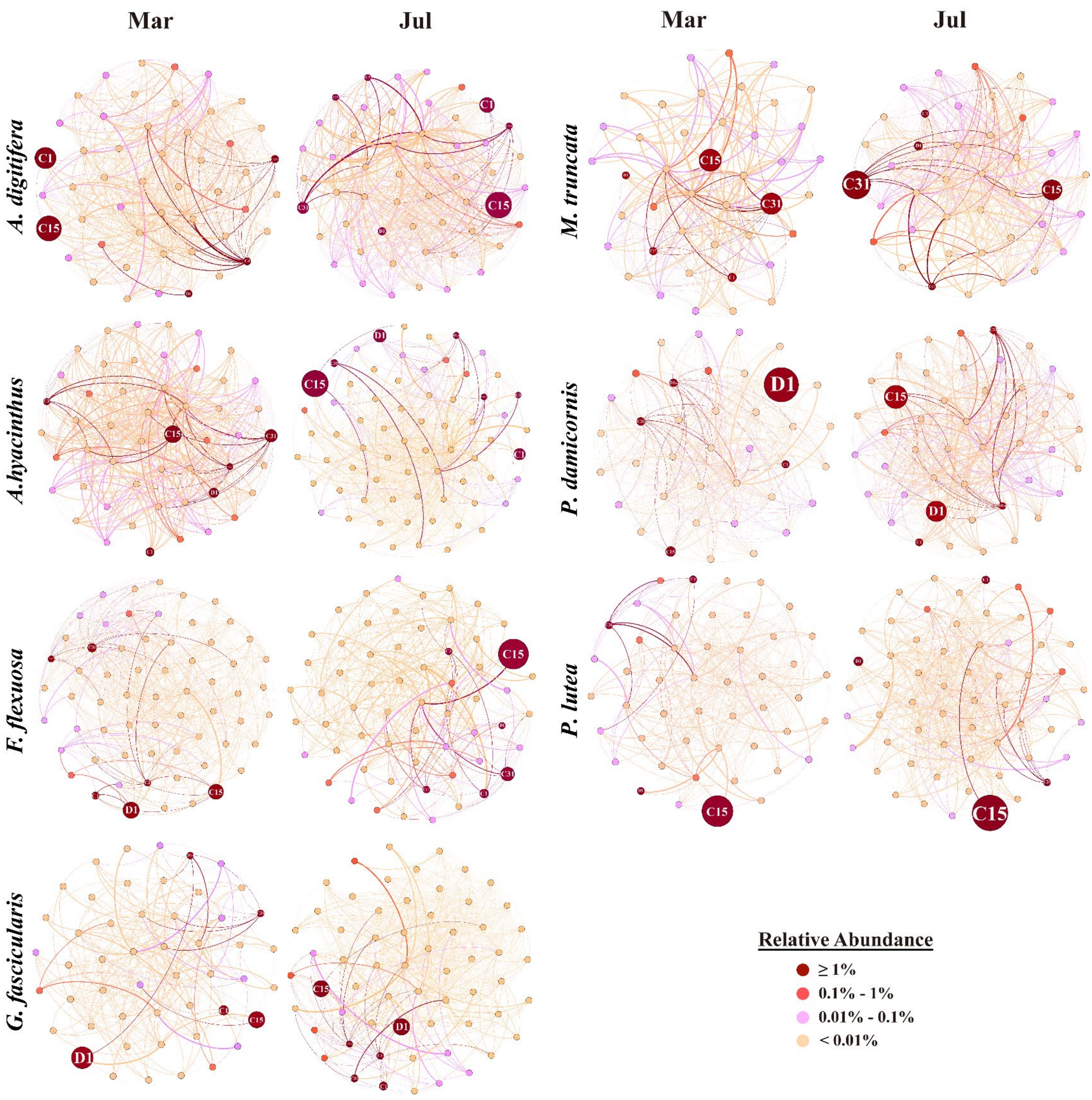

Fig. S8 The networks of Symbiodiniaceae in various coral species. The node size is proportional to the relative abundance of symbiont genotypes in the host corals. The edge width is equivalent to the edge betweenness values.

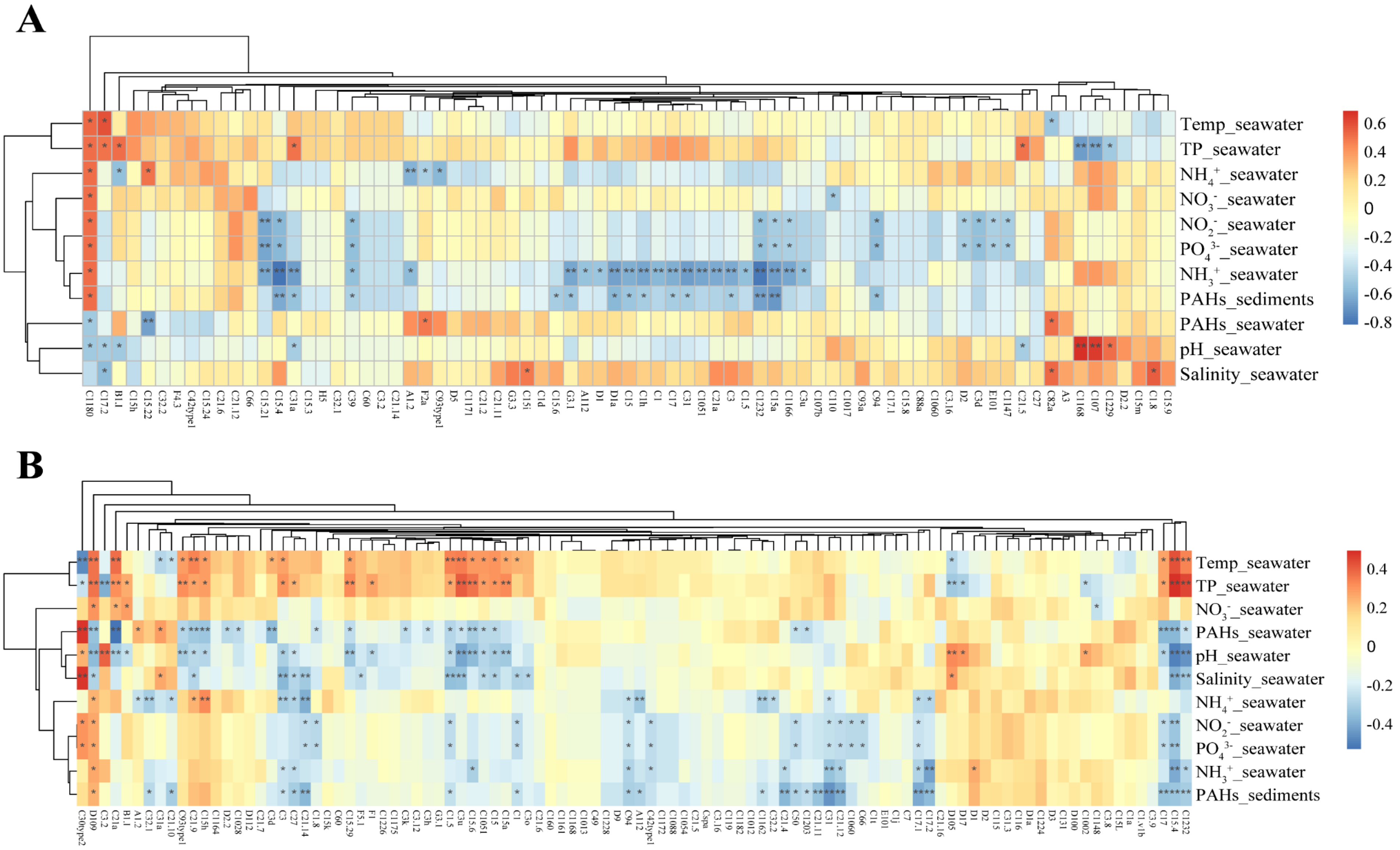

Fig. S9 Heat maps (DHCA analysis and Pearson correction analysis) for relationships between the environmental factors and the genotypes abundance in seawater (A) and corals (B) (*, $p < 0.05$; **, $p < 0.01$).

Supplementary Tables

Table S1 The mean month sea surface temperature (SST) in Hainan Island, China.

(Data from the website https://psl.noaa.gov/)

**Monthes**	**1891-1949**	**1950-1999**	**2000-2009**	**2010-2022**	**2017**
Jan	23.2±0.6	23.3±0.6	23.6±0.4	23.9±0.6	23.9
Feb	23.1±0.7	23.3±0.6	23.4±0.4	23.5±0.6	23.2
Mar	24.1±0.6	24.3±0.7	24.5±0.7	24.7±0.7	**24.7**
Apr	26.1±0.5	26.3±0.6	26.7±0.5	26.5±0.6	27.0
May	28.2±0.4	28.3±0.4	28.4±0.5	28.9±0.4	29.0
Jun	29±0.4	29.1±0.4	29.3±0.3	29.7±0.3	29.7
Jul	29±0.3	29.2±0.4	29.6±0.3	29.7±0.3	**29.5**
Aug	28.9±0.4	29.1±0.3	29.4±0.3	29.6±0.4	29.7
Sep	28.6±0.4	28.8±0.4	29.1±0.4	29.4±0.5	29.4
Oct	27.2±0.5	27.6±0.5	27.9±0.5	28.1±0.5	28.5
Nov	25.7±0.5	26±0.5	26.5±0.4	26.6±0.5	26.6
Dec	24.2±0.6	24.4±0.5	24.9±0.5	25.1±0.7	24.7

Table S2 Description for sampling stations and coral samples in this study.

**Sampling sites**	**Latitude**	**Longitude**	**Coral genus**	**Coral species**	**N**	**Sampling time**
Luhuitou	18°12'45.28"N	109°28'45.28"E	*Acropora*	*A. digitifera*	6	March & July
			Acropora	*A. hyacinthus*	6	March & July
			Favites	*F. flexuosa*	6	March & July
			Galaxea	*G. fascicularis*	6	March & July
			Montipora	*M. truncata*	6	March & July
			Porites	*P. lutea*	6	March & July
Phoenix Island	18°14'32.62"N	109°29'13.89"E	*Galaxea*	*G. fascicularis*	6	March & July
			Favites	*F. flexuosa*	6	March & July
			Pocillopora	*P. damicornis*	6	March & July
			Porites	*P. lutea*	6	March & July
			Montipora	*M. truncata*	6	March & July
Wuzhizhou Island	18°19'1.67"N	109°45'46.88"E	*Acropora*	*A. hyacinthus*	3	July
			Favites	*F. flexuosa*	6	March & July

N: The number of collected samples

Table S3 Summary for the genus level sequence number and relative abundance of Symbiodiniaceae in coral reefs in March and July.

Taxon	Abbreviation	Seawater				Corals			
		March		July		March		July	
		Sequence Number	Relative Abundance	Sequence Number	Relative Abundance	Sequence Number	Relative Abundance	Sequence Number	Relative Abundance
Cladocopium	C	197881	(32.86%)	327942	(47.2%)	1760628	(63.6%)	2148841	(82.71%)
Durusdinium	D	7213	(1.2%)	12593	(1.84%)	886661	(30.49%)	480536	(16.53%)
Breviolum	B	643	(< 0.01%)	736	(< 0.01%)	90	(< 0.01%)	59	(< 0.01%)
Effrenium	E	1	(< 0.01%)	2	(< 0.01%)	831	(< 0.01%)	150	(< 0.01%)
Symbiodinium	A	27	(< 0.01%)	48	(< 0.01%)	538	(< 0.01%)	212	(< 0.01%)
Gerakladium	G	77	(< 0.01%)	212	(< 0.01%)	11	(< 0.01%)	63	(< 0.01%)
Fugacium	F	142	(< 0.01%)	92	(< 0.01%)	9	(< 0.01%)	53	(< 0.01%)
Clade H	H	0	0	2	(< 0.02%)	0	0	0	0
Others		402545	(65.77%)	366045	(50.76%)	172056	(5.86%)	20498	(< 0.01%)
**SUM**		608529		707672		2820824		2650412	

Table S4 Alpha diversity of Symbiodiniaceae in seawater and corals.

Samples	Chao1		Shannon	
	March	July	March	July
Seawater	1656.3±373.6	1691.6±442.5	4.1±0.8	3.7±0.9
All corals	1264.3±277.4	1548.5±290	2±0.6	2.1±0.6
A. digitifera	1419.5±175.9	2043.7±115.2	2.8±0.2	3.3±0.2
A. hyacinthus	1622±89.7	1879.3±209.9	2.8±0.2	2.7±0.2
F. flexuosa	1269.3±308.7	1380.6±184.4	1.9±0.7	2±0.5
G. fascicularis	1387.5±151.3	1466.8±132.9	2.2±0.3	2.1±0.2
M. truncata	1078.2±88.1	1292.8±107.4	1.8±0.1	2±0.1
P. damicornis	1379.6±110.1	1611.8±167.4	1.5±0.2	1.8±0.5
P. lutea	912.4±82.3	1400.1±214.5	1.3±0.3	1.5±0.3

Table S5 Summary for the genus levels relative abundance of Symbiodiniaceae in seawater and various coral species at the sampling sites.

Sampling Sites	Samples	Months	Taxon								
			Cladocopium	*Durusdinium*	*Breviolum*	*Effrenium*	*Symbiodinium*	*Gerakladium*	*Fugacium*	Clade H	Others
**Luhuitou**	**Seawater**	**Mar**	68.41%	2.67%	0.35%	0	0.01%	0.04%	0.08%	0	28.43%
		**Jul**	49.03%	2.24%	0.42%	0	0.02%	0.05%	0.06%	< 0.01%	48.19%
**Phoenix Island**	**Seawater**	**Mar**	8.36%	0.24%	0	0	0	< 0.01%	0	0	91.40%
		**Jul**	20.71%	0.61%	< 0.01%	0	0	< 0.01%	< 0.01%	0	78.67%
**Wuzhizhou Island**	**Seawater**	**Mar**	21.81%	0.70%	0	< 0.01%	0	< 0.01%	0	0	77.49%
		**Jul**	71.86%	2.65%	< 0.01%	< 0.01%	< 0.01%	0.04%	< 0.01%	0	25.43%
**Luhuitou**	***A. digitifera***	**Mar**	97.42%	2.10%	< 0.01%	< 0.01%	0.03%	< 0.01%	0	0	0.44%
		**Jul**	94.08%	5.18%	0	< 0.01%	0.06%	< 0.01%	< 0.01%	0	0.66%
	A. hyacinthus	**Mar**	80.92%	14.28%	< 0.01%	0.31%	0.14%	< 0.01%	< 0.01%	0	4.35%
		**Jul**	84.45%	14.63%	< 0.01%	< 0.01%	< 0.01%	< 0.01%	< 0.01%	0	0.89%
	F. flexuosa	**Mar**	16.21%	83.62%	0.02%	< 0.01%	< 0.01%	0	0	0	0.14%
		**Jul**	97.23%	2.73%	< 0.01%	< 0.01%	0	0	< 0.01%	0	0.04%
	G. fascicularis	**Mar**	24.75%	67.20%	< 0.01%	< 0.01%	< 0.01%	0	0	0	8.05%
		**Jul**	88.00%	11.35%	0	< 0.01%	< 0.01%	< 0.01%	< 0.01%	0	0.64%
	M. truncata	**Mar**	94.77%	4.99%	< 0.01%	< 0.01%	< 0.01%	0	< 0.01%	0	0.23%
		**Jul**	98.06%	1.18%	< 0.01%	0	0.01%	0	< 0.01%	0	0.74%
	P. lutea	**Mar**	93.77%	2.83%	< 0.01%	0	< 0.01%	< 0.01%	0	0	3.39%
		**Jul**	91.43%	8.30%	< 0.01%	< 0.01%	< 0.01%	< 0.01%	0	0	0.26%
**Phoenix Island**	***F. flexuosa***	**Mar**	81.94%	17.84%	< 0.01%	< 0.01%	< 0.01%	< 0.01%	0	0	0.21%
		**Jul**	98.93%	0.99%	< 0.01%	< 0.01%	< 0.01%	< 0.01%	0	0	0.08%
	G. fascicularis	**Mar**	63.94%	30.34%	0	< 0.01%	0.01%	< 0.01%	0	0	5.71%
		**Jul**	24.97%	71.42%	< 0.01%	< 0.01%	0.03%	0	< 0.01%	0	3.58%
	P. damicornis	**Mar**	15.85%	83.98%	0	< 0.01%	< 0.01%	0	0	0	0.16%
		**Jul**	56.03%	43.58%	< 0.01%	< 0.01%	< 0.01%	< 0.01%	< 0.01%	0	0.37%
	P. lutea	**Mar**	56.38%	2.21%	0	< 0.01%	0.01%	< 0.01%	0	0	41.40%
		**Jul**	89.16%	10.07%	< 0.01%	< 0.01%	0	< 0.01%	< 0.01%	0	0.76%
**Wuzhizhou Island**	***A. hyacinthus***	**Jul**	72.11%	27.14%	< 0.01%	0.04%	0	< 0.01%	< 0.01%	0	0.69%
	F. flexuosa	**Mar**	73.68%	25.96%	< 0.01%	0	< 0.01%	< 0.01%	0	0	0.36%
		**Jul**	98.08%	1.79%	0	< 0.01%	< 0.01%	0	0	0	0.13%

Table S6 Symbiodiniaceae genotypes with significant differences in the relative abundance between March and July.

Samples	Taxon	Relative Abundance		p-value
		**Mar**	**Jul**	
Seawater	C17.2	< 0.01%	< 0.01%	0.03
	C31a	< 0.01%	< 0.01%	0.04
Corals	C1	8.71%	9.34%	0.04
	C1.5	0.10%	0.13%	< 0.01
	C1.8	< 0.01%	< 0.01%	0.03
	C1051	0.02%	0.04%	< 0.01
	C1232	0.32%	0.40%	< 0.01
	C15	40.70%	57.44%	< 0.01
	C15.29	< 0.01%	< 0.01%	0.01
	C15.4	< 0.01%	0.01%	< 0.01
	C15.6	< 0.01%	< 0.01%	< 0.01
	C15a	0.02%	0.03%	< 0.01
	C17	0.73%	1.26%	< 0.01
	C21a	0.08%	0.15%	< 0.01
	C27	< 0.01%	< 0.01%	0.03
	C3	0.84%	1.35%	0.01
	C3.2	< 0.01%	0	0.03
	C30type2	< 0.01%	< 0.01%	< 0.01
	C31	11.85%	12.26%	0.04
	C3d	0.02%	0.02%	0.04
	C3k	< 0.01%	< 0.01%	0.05
	C3o	< 0.01%	< 0.01%	0.04
	C3u	0.05%	0.13%	< 0.01
	D105	< 0.01%	0	0.02
	D109	0	< 0.01%	0.05
	D17	< 0.01%	< 0.01%	0.03

Table S7 Summary of the sequence number and relative abundance of Symbiodiniaceae genotypes in seawater and corals in March and July.

Taxon	Sequence Number				Relative Abundance			
	Seawater		Corals		Seawater		Corals	
	Mar	Jul	Mar	Jul	Mar	Jul	Mar	Jul
C15	151744	246915	1144813	1512366	25.05%	35.50%	40.70%	57.44%
D1	145	214	859865	453020	0.03%	0.03%	29.56%	15.54%
C31	5385	10737	326344	316515	0.89%	1.53%	11.85%	12.26%
C1	37885	65215	225623	225371	6.44%	9.44%	8.71%	9.34%
D1a	7061	12372	26285	27200	1.18%	1.80%	0.91%	0.98%
C3	28	36	24604	36016	< 0.01%	< 0.01%	0.84%	1.35%
C17	524	1004	20395	32967	0.09%	0.14%	0.73%	1.26%
C1232	205	596	7008	8776	0.03%	0.08%	0.32%	0.40%
C21a	286	335	2159	4077	0.05%	0.05%	0.08%	0.15%
C1.5	212	252	2511	3167	0.04%	0.04%	0.10%	0.13%
C3u	11	11	1497	3541	< 0.01%	< 0.01%	0.05%	0.13%
C15a	749	1243	435	684	0.12%	0.18%	0.02%	0.03%
C1051	325	488	692	963	0.06%	0.07%	0.02%	0.04%
C17.1	13	12	1241	1140	< 0.01%	< 0.01%	0.04%	0.05%
B1.I	643	736	90	59	0.12%	0.14%	< 0.01%	< 0.01%
C1203	0	0	701	486	0	0	0.03%	0.02%
E101	1	2	831	150	< 0.01%	< 0.01%	0.03%	< 0.01%
C3d	4	8	451	418	< 0.01%	< 0.01%	0.02%	0.02%
C31a	3	14	469	324	< 0.01%	< 0.01%	0.02%	0.01%
A1.2	21	21	537	203	< 0.01%	< 0.01%	0.02%	< 0.01%
D2	2	2	459	294	< 0.01%	< 0.01%	0.02%	0.01%
C21.4	0	0	419	338	0	0	0.02%	0.01%
C3.9	0	0	319	178	0	0	0.01%	< 0.01%
C21.12	1	1	177	250	< 0.01%	< 0.01%	< 0.01%	< 0.01%
C27	113	278	3	14	0.02%	0.04%	< 0.01%	< 0.01%
C1h	127	260	0	0	0.02%	0.04%	0	0
C15.4	16	38	61	268	< 0.01%	< 0.01%	< 0.01%	0.01%
G3.1	75	208	11	63	0.01%	0.03%	< 0.01%	< 0.01%
C15.6	64	85	29	144	< 0.01%	0.01%	< 0.01%	< 0.01%
C21.6	2	4	121	131	< 0.01%	< 0.01%	< 0.01%	< 0.01%
C42type1	40	202	0	4	< 0.01%	0.03%	0	< 0.01%
F2a	142	87	0	0	0.03%	0.02%	0	0
C21.11	20	15	83	100	< 0.01%	< 0.01%	< 0.01%	< 0.01%
C17.2	2	17	70	123	< 0.01%	< 0.01%	< 0.01%	< 0.01%
C32.1	2	4	69	83	< 0.01%	< 0.01%	< 0.01%	< 0.01%
C50	0	0	74	50	0	0	< 0.01%	< 0.01%
C21.14	0	2	37	72	0	< 0.01%	< 0.01%	< 0.01%
C1226	0	0	33	36	0	0	< 0.01%	< 0.01%
C21.5	8	26	8	15	< 0.01%	< 0.01%	< 0.01%	< 0.01%
F1	0	0	9	45	0	0	< 0.01%	< 0.01%
C30type2	0	0	42	1	0	0	< 0.01%	< 0.01%
C93type1	13	19	1	8	< 0.01%	< 0.01%	< 0.01%	< 0.01%
D17	0	0	38	3	0	0	< 0.01%	< 0.01%
A112	4	27	1	9	< 0.01%	< 0.01%	< 0.01%	< 0.01%
C1.8	6	4	5	23	< 0.01%	< 0.01%	< 0.01%	< 0.01%
C107b	15	18	0	0	< 0.01%	< 0.01%	0	0
C3o	0	0	9	21	0	0	< 0.01%	< 0.01%

Taxon	Sequence Number				Relative Abundance			
	Seawater		Corals		Seawater		Corals	
	Mar	Jul	Mar	Jul	Mar	Jul	Mar	Jul
C66	10	12	2	3	< 0.01%	< 0.01%	< 0.01%	< 0.01%
C21.10	0	0	15	11	0	0	< 0.01%	< 0.01%
C15i	14	12	0	0	< 0.01%	< 0.01%	0	0
C1060	4	6	8	6	< 0.01%	< 0.01%	< 0.01%	< 0.01%
C94	6	10	1	5	< 0.01%	< 0.01%	< 0.01%	< 0.01%
C3h	0	0	4	16	0	0	< 0.01%	< 0.01%
C60	0	2	6	11	0	< 0.01%	< 0.01%	< 0.01%
C15.29	0	0	2	13	0	0	< 0.01%	< 0.01%
C3.2	0	2	12	0	0	< 0.01%	< 0.01%	0
C1161	0	0	7	6	0	0	< 0.01%	< 0.01%
C32.2	1	3	3	5	< 0.01%	< 0.01%	< 0.01%	< 0.01%
C3k	0	0	2	10	0	0	< 0.01%	< 0.01%
C21.9	0	0	2	9	0	0	< 0.01%	< 0.01%
Cspa	0	0	3	7	0	0	< 0.01%	< 0.01%
C93a	5	5	0	0	< 0.01%	< 0.01%	0	0
D105	0	0	9	0	0	0	< 0.01%	0
F5.1	0	0	0	8	0	0	0	< 0.01%
C15.22	1	7	0	0	< 0.01%	< 0.01%	0	0
C15.24	2	6	0	0	< 0.01%	< 0.01%	0	0
D5	3	5	0	0	< 0.01%	< 0.01%	0	0
C1.v1b	0	0	4	3	0	0	< 0.01%	< 0.01%
D2.2	2	0	1	4	< 0.01%	0	< 0.01%	< 0.01%
D109	0	0	0	7	0	0	0	< 0.01%
C1162	0	0	3	4	0	0	< 0.01%	< 0.01%
C21.16	0	0	4	3	0	0	< 0.01%	< 0.01%
C15h	0	3	0	4	0	< 0.01%	0	< 0.01%
C1168	5	0	1	1	< 0.01%	0	< 0.01%	< 0.01%
C115	0	0	2	5	0	0	< 0.01%	< 0.01%
C1166	2	5	0	0	< 0.01%	< 0.01%	0	0
C1d	3	4	0	0	< 0.01%	< 0.01%	0	0
C131	0	0	4	2	0	0	< 0.01%	< 0.01%
C1002	0	0	6	0	0	0	< 0.01%	0
C3.16	1	2	1	2	< 0.01%	< 0.01%	< 0.01%	< 0.01%
G3.3	2	4	0	0	< 0.01%	< 0.01%	0	0
C7	0	0	3	2	0	0	< 0.01%	< 0.01%
C21.7	0	0	2	3	0	0	< 0.01%	< 0.01%
C160	0	0	2	3	0	0	< 0.01%	< 0.01%
C15L	0	0	4	1	0	0	<0.01%	< 0.01%
C110	3	2	0	0	< 0.01%	< 0.01%	0	0
C107	5	0	0	0	< 0.01%	0	0	0
C1017	3	2	0	0	< 0.01%	< 0.01%	0	0
F4.3	0	5	0	0	0	< 0.01%	0	0
C15k	0	0	1	3	0	0	< 0.01%	< 0.01%
C1164	0	0	1	3	0	0	< 0.01%	< 0.01%
C116	0	0	3	1	0	0	< 0.01%	< 0.01%
D100	0	0	2	2	0	0	< 0.01%	< 0.01%
C1028	0	0	0	4	0	0	0	< 0.01%
D3	0	0	2	2	0	0	< 0.01%	< 0.01%
C1229	4	0	0	0	< 0.01%	0	0	0
C1j	0	0	2	1	0	0	< 0.01%	< 0.01%
C1054	0	0	1	2	0	0	< 0.01%	< 0.01%

Taxon	Sequence Number				Relative Abundance			
	Seawater		Corals		Seawater		Corals	
	Mar	Jul	Mar	Jul	Mar	Jul	Mar	Jul
C31.3	0	0	2	1	0	0	< 0.01%	< 0.01%
C3.12	0	0	0	3	0	0	0	< 0.01%
C1175	0	0	0	3	0	0	0	< 0.01%
C1012	0	0	1	2	0	0	< 0.01%	< 0.01%
C1180	0	3	0	0	0	< 0.01%	0	0
C15.21	0	3	0	0	0	< 0.01%	0	0
C15.9	2	1	0	0	< 0.01%	< 0.01%	0	0
C39	0	3	0	0	0	< 0.01%	0	0
C1147	1	2	0	0	< 0.01%	< 0.01%	0	0
C1t	0	0	0	2	0	0	0	< 0.01%
C1013	0	0	1	1	0	0	< 0.01%	< 0.01%
C3.8	0	0	2	0	0	0	< 0.01%	0
C1172	0	0	0	2	0	0	0	< 0.01%
C1228	0	0	0	2	0	0	0	< 0.01%
C1088	0	0	0	2	0	0	0	< 0.01%
C119	0	0	1	1	0	0	< 0.01%	< 0.01%
C49	0	0	1	1	0	0	< 0.01%	< 0.01%
C1182	0	0	1	1	0	0	< 0.01%	< 0.01%
D112	0	0	0	2	0	0	0	< 0.01%
C1224	0	0	1	1	0	0	< 0.01%	< 0.01%
C1148	0	0	2	0	0	0	< 0.01%	0
D9	0	0	0	2	0	0	0	< 0.01%
C1a	0	0	2	0	0	0	< 0.01%	0
C15.8	0	2	0	0	0	< 0.01%	0	0
A3	2	0	0	0	< 0.01%	0	0	0
C88a	0	2	0	0	0	< 0.01%	0	0
C15.3	0	2	0	0	0	< 0.01%	0	0
C82a	2	0	0	0	< 0.01%	0	0	0
C1171	1	1	0	0	< 0.01%	< 0.01%	0	0
H5	0	2	0	0	0	< 0.01%	0	0
C21.2	1	1	0	0	< 0.01%	< 0.01%	0	0
C15m	2	0	0	0	< 0.01%	0	0	0
No Blast	402545	366045	172056	20498	65.77%	50.76%	5.86%	0.74%
SUM	608529	707672	2820824	2650412	25.05%	35.50%	40.70%	57.44%

Table S8 The various genotypes relative abundance of Symbiodiniaceae within seawater and coral species samples at the sampling sites.

Taxon	Corals_March																					
	Luhuitou																		Phoenix			
	A. digitifera_1	*A. digitifera_2*	*A. digitifera_3*	*A. hyacinthus_1*	*A. hyacinthus_2*	*A. hyacinthus_3*	*F. flexuosa_1*	*F. flexuosa_2*	*F. flexuosa_3*	*G. fascicularis_1*	*G. fascicularis_2*	*G. fascicularis_3*	*M. truncata_1*	*M. truncata_2*	*M. truncata_3*	*P. lutea_1*	*P. lutea_2*	*P. lutea_3*	*F. flexuosa_1*	*F. flexuosa_2*	*F. flexuosa_3*	*G. fascicularis_1*
C1	38.67%	40.31%	40.77%	8.56%	11.38%	7.59%	1.32%	2.06%	1.91%	3.55%	3.68%	3.37%	3.09%	2.60%	2.07%	1.94%	2.00%	1.88%	8.39%	9.04%	9.56%	16.02%
D1a	0.63%	0.12%	0.13%	1.42%	0.71%	0.61%	1.27%	1.20%	1.21%	1.54%	1.90%	1.68%	0.29%	0.19%	0.16%	0.15%	0.14%	0.10%	0.63%	0.45%	0.60%	0.92%
D1	3.94%	0.87%	0.61%	21.89%	10.61%	7.56%	87.03%	80.31%	79.83%	66.36%	62.83%	66.98%	6.67%	4.44%	3.20%	3.12%	2.61%	2.36%	20.14%	16.28%	15.42%	32.77%
C21a	<0.01%	<0.01%	0.01%	0.04%	0.05%	0.07%	<0.01%	0.02%	0.01%	0.01%	0.03%	0.02%	0.05%	0.07%	0.09%	0.02%	0.03%	0.03%	0.02%	<0.01%	0.01%	<0.01%
C17	0.25%	0.28%	0.23%	0.90%	1.30%	1.57%	0.11%	0.17%	0.20%	0.23%	0.28%	0.28%	1.57%	1.67%	1.52%	0.22%	0.31%	0.30%	0.17%	0.23%	0.29%	0.16%
C3u	0.06%	0.07%	0.03%	0.08%	0.05%	0.07%	0.02%	0.05%	0.05%	0.02%	0.03%	0.02%	0.02%	0.02%	<0.01%	0.08%	0.06%	0.05%	0.23%	0.22%	0.25%	0.02%
C93typ	0	0	0	0	0	0	0	0	0	0	0	0	0	0	0	<0.01%	0	0	0	0	0	0
C1232	3.54%	2.15%	2.99%	0.40%	0.25%	0.15%	0.04%	0.08%	0.01%	0.06%	0.02%	0.02%	0.08%	0.05%	0.05%	0.05%	0.03%	0.01%	0.04%	0.05%	0.03%	0.05%
C1203	<0.01%	<0.01%	<0.01%	0.01%	<0.01%	0.02%	<0.01%	<0.01%	<0.01%	<0.01%	0.02%	0.02%	0.04%	0.03%	0.03%	<0.01%	<0.01%	0.01%	<0.01%	0	<0.01%	0
C17.1	<0.01%	0.01%	0.01%	0.10%	0.14%	0.20%	<0.01%	<0.01%	<0.01%	<0.01%	0.02%	0.01%	0.20%	0.21%	0.17%	<0.01%	<0.01%	0.01%	<0.01%	<0.01%	<0.01%	<0.01%
C15a	<0.01%	<0.01%	<0.01%	0.03%	0.04%	0.03%	<0.01%	<0.01%	<0.01%	0.01%	0.01%	<0.01%	0	0	<0.01%	0.04%	0.06%	0.06%	<0.01%	<0.01%	<0.01%	<0.01%
C31	2.13%	3.78%	2.20%	17.61%	24.47%	33.21%	1.55%	2.67%	3.35%	2.59%	3.51%	3.94%	51.63%	55.56%	57.06%	3.53%	4.98%	5.47%	1.36%	1.79%	2.44%	0.87%
D2	<0.01%	<0.01%	<0.01%	0.01%	<0.01%	<0.01%	<0.01%	<0.01%	<0.01%	0.06%	0.12%	0.10%	<0.01%	<0.01%	<0.01%	<0.01%	0	0	0.01%	<0.01%	<0.01%	0.02%
C15	48.26%	51.06%	51.92%	40.56%	45.23%	43.37%	8.01%	12.47%	12.42%	17.47%	18.15%	15.94%	35.33%	34.22%	34.85%	86.80%	85.98%	86.14%	64.21%	67.60%	65.81%	42.12%
C1051	0.04%	0.04%	0.04%	0.05%	0.05%	0.05%	0.01%	0.02%	0.01%	0.02%	0.02%	0.01%	0.01%	<0.01%	<0.01%	0.01%	0.02%	0.01%	0.04%	0.04%	0.06%	0.02%
C1.5	0.93%	0.28%	0.26%	0.12%	0.11%	0.11%	0.02%	0.03%	0.03%	0.02%	0.04%	0.04%	0.04%	0.03%	0.02%	0.03%	0.03%	0.03%	0.15%	0.14%	0.22%	0.10%
C32.2	0	0	0	0	0	<0.01%	0	0	0	<0.01%	0	0	<0.01%	0	0	0	0	0	0	0	0	0
C3d	<0.01%	<0.01%	<0.01%	0.01%	0.03%	0.02%	0	<0.01%	<0.01%	<0.01%	<0.01%	<0.01%	0.02%	0.03%	0.03%	<0.01%	<0.01%	<0.01%	<0.01%	<0.01%	<0.01%	<0.01%
C17.2	0	<0.01%	<0.01%	<0.01%	0.01%	<0.01%	<0.01%	0	<0.01%	0	<0.01%	<0.01%	<0.01%	0.02%	0.01%	0	<0.01%	0	0	0	<0.01%	0
C15.6	<0.01%	0	0	<0.01%	<0.01%	<0.01%	0	0	0	0	0	<0.01%	0	<0.01%	0	<0.01%	<0.01%	<0.01%	0	<0.01%	0	<0.01%
C3	0.36%	0.57%	0.44%	1.62%	1.25%	1.36%	0.45%	0.71%	0.69%	0.20%	0.24%	0.25%	0.47%	0.43%	0.34%	0.30%	0.34%	0.32%	4.28%	3.97%	5.06%	0.43%
C1.v1b	0	0	0	0	<0.01%	0	0	0	0	0	0	0	0	<0.01%	0	0	0	<0.01%	0	0	0	0
C15k	0	0	0	0	<0.01%	0	0	0	0	0	0	0	0	0	0	0	0	0	0	0	0	0
C3.2	0	0	0	0	0	0	0	0	0	<0.01%	0	0	0	0	0	0	0	0	0	0	0	0
C21.6	0.01%	<0.01%	0.01%	<0.01%	<0.01%	<0.01%	0	0	<0.01%	<0.01%	<0.01%	<0.01%	<0.01%	<0.01%	<0.01%	<0.01%	0	0	0	<0.01%	0	0
C1.8	0	0	0	0	0	0	0	0	0	0	<0.01%	0	0	0	0	0	0	0	<0.01%	0	0	0
C1t	0	0	0	0	0	0	0	0	0	0	0	0	0	0	0	0	0	0	0	0	0	0
C31a	<0.01%	0.01%	<0.01%	0.04%	0.05%	0.07%	<0.01%	<0.01%	<0.01%	<0.01%	<0.01%	<0.01%	0.06%	0.09%	0.11%	<0.01%	<0.01%	<0.01%	<0.01%	0	<0.01%	<0.01%
C21.11	0	<0.01%	0	0.01%	<0.01%	<0.01%	0	<0.01%	0	<0.01%	<0.01%	<0.01%	<0.01%	<0.01%	<0.01%	<0.01%	<0.01%	0	<0.01%	0	<0.01%	0
C3o	0	<0.01%	<0.01%	0	0	0	0	<0.01%	0	0	0	0	0	0	0	0	0	0	0	<0.01%	<0.01%	0
C21.12	0.01%	0.01%	<0.01%	<0.01%	0.01%	0.02%	<0.01%	<0.01%	<0.01%	<0.01%	<0.01%	0	0.02%	0.01%	0.02%	<0.01%	<0.01%	<0.01%	0	<0.01%	<0.01%	<0.01%
D17	0	0	0	<0.01%	0	0	0	0	0	<0.01%	<0.01%	<0.01%	0	0	0	0	0	0	0	0	0	<0.01%
C66	0	0	0	0	0	0	0	0	0	0	0	0	0	0	0	0	0	0	0	0	0	0
C3k	0	0	<0.01%	0	0	0	0	0	0	0	0	0	0	0	0	0	0	0	0	0	0	0
C21.5	0	<0.01%	0	0	0	0	0	0	0	0	0	0	<0.01%	0	0	0	<0.01%	0	0	0	0	0
C21.4	<0.01%	<0.01%	<0.01%	0.03%	0.03%	0.03%	<0.01%	<0.01%	<0.01%	<0.01%	<0.01%	<0.01%	0.04%	0.04%	0.05%	<0.01%	0.01%	<0.01%	0	0	<0.01%	0
E101	<0.01%	<0.01%	<0.01%	0.69%	0.10%	0.13%	0	<0.01%	0	<0.01%	0	0	<0.01%	<0.01%	<0.01%	0	0	0	<0.01%	<0.01%	<0.01%	<0.01%
C1j	0	0	<0.01%	0	0	0	0	0	0	0	0	0	0	0	0	0	0	0	0	0	0	0
F5.1	0	0	0	0	0	0	0	0	0	0	0	0	0	0	0	0	0	0	0	0	0	0

Table S9 The node betweenness centrality values of the network based on Symbiodiniaceae community in seawater, corals, and various coral species.

Items	Months	Relative Abundance			
		<0.01%	0.01%-0.1%	0.1%-1%	≥1%
Seawater-Corals	Mar	3.259	3.628	4.683	13.216
	Jul	4.311	2.412	3.266	23.885
Seawater	Mar	7.204	0.179	0.642	0
	Jul	8.152	0.243	0.080	0
Corals	Mar	15.276	1.489	0.631	0.540
	Jul	10.298	0.688	0.278	0.380
A. digitifera	Mar	4.417	0.132	0.134	0.086
	Jul	0.536	0.005	0	0.001
A. hyacinthus	Mar	0.544	0.009	0.013	0.006
	Jul	0.825	0.040	0.020	0.011
F. flexuosa	Mar	0.671	0.076	0.006	0.053
	Jul	0.883	0.102	0.071	0.040
G. fascicularis	Mar	0.937	0.045	0.001	0.021
	Jul	0.659	0.052	0.010	0.034
M. truncata	Mar	0.502	0.007	0.003	0.003
	Jul	0.680	0.015	0.005	0.009
P. damicornis	Mar	0.517	0.003	0.001	0.004
	Jul	0.525	0.020	0.001	0.005
P. lutea	Mar	0.824	0.045	0.008	0.033
	Jul	0.802	0.008	0.011	0.003

Table S10 The network complexity and stability of Symbiodiniaceae in seawater, corals, and various coral species.

Samples	Complexity		Vulnerability		Compositional Stability
	Mar	Jul	Mar	Jul	
Seawater	6.136	5.887	0.034	0.038	0.867
Corals	6.414	4.318	0.012	0.018	0.919
A. digitifera	12.694	12.192	0.008	0.009	0.801
A. hyacinthus	11.275	9.681	0.012	0.035	0.771
F. flexuosa	10.413	6.217	0.010	0.021	0.798
G. fascicularis	6.160	10.951	0.022	0.017	0.943
M. truncata	10.220	6.829	0.012	0.021	0.923
P. damicornis	9.316	10.652	0.034	0.013	0.761
P. lutea	7.063	8.847	0.022	0.017	0.966

Table S11 Spatial and temporal distribution of the environmental factors in Sanya coral reefs, Hainan, China.

Items	March			July		
	**Luhuitou**	**Phoenix Island**	**Wuzhizhou Island**	**Luhuitou**	**Phoenix Island**	**Wuzhizhou Island**
SST (°C)	23.6±0.9	24.6±0.7	24.6±0.9	**30.4±0.4**	**30.6±0.3**	**28.2±0.4**
pH_seawater	**8.0±0.2**	**8.1±0.1**	**8.1±0.1**	7.9±0.1	7.9±0.1	7.9±0.1
Salinity_seawater (‰)	**37±0.1**	**33±0.1**	**36±0.1**	31±0.1	31±0.1	34±0.1
NO_2^-_seawater (μmol·L^{-1})	**0.3±0**	0.2±0	0.1±0	0.1±0	**0.9±0**	0.1±0
NO_3^-_seawater (μmol·L^{-1})	**1.3±0.3**	0.6±0.1	**3.8±0.5**	0.7±0.1	**11.2±0.2**	0.6±0
NH_3^+_seawater (μmol·L^{-1})	**0.8±0.3**	0.9±0.4	**2.2±0.7**	0.3±0.3	**7.1±0.5**	0.1±0.5
NH_4^+_seawater (μmol·L^{-1})	0.1±0.3	0.7±0.1	**2.1±0.3**	0.1±0	**22±0.7**	0.7±0.1
PO_4^{3-}_seawater (μmol·L^{-1})	**0.3±0**	0.3±0.3	**0.3±0**	0.2±0	**1.0±0**	0.2±0
TP_seawater (μmol·L^{-1})	**0.7±0**	0.6±0.6	**0.5±0**	0.3±0	**1.4±0**	0.3±0
∑PAHs_seawater (ng·L^{-1})	**372.8±306.3**	**294.1±70.7**	**240.6±57.9**	282.8±7.6	217.6±33.6	234.4±73.5
∑PAHs_sediments (ng·g^{-1} dw)	**351.9±144.2**	385.8±220	**328.4±191.5**	204.2±50.3	**387.5±151.3**	312.8±95.7

∑PAHs: The sixteen polycyclic aromatic hydrocarbons

The data about ∑PAHs concentrations of seawater and sediments, and physical/chemical parameters in July were published [3,4]

Table S12 The decision curve analysis results for Symbiodiniaceae composition in seawater and corals.

Items	DCA1	DCA2	DCA3	DCA4
Axis lengths (seawater)	1.42	0.30	0.27	0.27
Axis lengths (corals)	2.02	1.75	1.50	1.16

References

1. *GB/T 12763.4-2007*; Specification of Oceanographic survey-part 4: survey of chemical parameters in sea water. Standards Press of China: Beijing, China, 2007. (In Chinese)
2. Yang, T.; Cheng, H.; Wang, H.; Drews, M.; Li, S.; Huang, W.; Zhou, H.; Chen, C.M.; Diao, X. Comparative study of polycyclic aromatic hydrocarbons (PAHs) and heavy metals (HMs) in corals, surrounding sediments and surface water at the Dazhou Island, China. *Chemosphere* **2019**, *218*, 157-168, doi:10.1016/j.chemosphere.2018.11.063.
3. Yang, T.; Diao, X.; Cheng, H.; Wang, H.; Zhou, H.; Zhao, H.; Chen, C.M. Comparative study of polycyclic aromatic hydrocarbons (PAHs) and heavy metals (HMs) in corals, sediments and seawater from coral reefs of Hainan, China. *Environmental pollution* **2020**, *264*, 114719, doi:10.1016/j.envpol.2020.114719.
4. Xiang, N.; Jiang, C.; Yang, T.; Li, P.; Wang, H.; Xie, Y.; Li, S.; Zhou, H.; Diao, X. Occurrence and distribution of Polycyclic aromatic hydrocarbons (PAHs) in seawater, sediments and corals from Hainan Island, China. *Ecotoxicology and environmental safety* **2018**, *152*, 8-15, doi:10.1016/j.ecoenv.2018.01.006.
